# A newly identified glycosyltransferase AsRCOM provides resistance to purple curl leaf disease in agave

**DOI:** 10.1186/s12864-023-09700-y

**Published:** 2023-11-07

**Authors:** Zhiwei Lu, Xiaowan Hou, Zhi Ke, Yanmei Zhang, ZiPing Yang, Wenzhao Zhou

**Affiliations:** 1 Zhanjiang City Key Laboratory for Tropical Crops Genetic Improvement, South Subtropical Crops Institute, Chinese Academy of Tropical Agricultural Sciences, Zhanjiang, Guangdong 524091 China; 2 State Key Laboratory of Plant Physiology and Biochemistry, College of Biological Sciences, China Agricultural University, Beijing, 100193 China; 3 Institute of Crop Science, Key Laboratory of Biology and Genetic Improvement of Triticeae Crops, Chinese Academy of Agricultural Sciences (CAAS), National Key Facility for Crop Gene Resources and Genetic Improvement, Ministry of Agriculture, Beijing, 100081 China; 4 Key Laboratory for Postharvest Physiology and Technology of Tropical Horticultural Products of Hainan Province, South Subtropical Crops Research Institute, Chinese Academy of Tropical Agricultural Sciences, Zhanjiang, Guangdong 524091 China

**Keywords:** Agave, Resistant mechanism, *AsRCOM* gene, Hypersensitive response

## Abstract

**Background:**

Purple curl leaf disease brings a significant threat to the development of agave industry, the underlying mechanism of disease-resistant *Agave sisalana*. hybrid 11648 (*A*. H11648R) is still unknown.

**Results:**

To excavate the crucial disease-resistant genes against purple curl leaf disease, we performed an RNA-seq analysis for *A*.H11648R and *A*.H11648 during different stages of purple curl leaf disease. The DEGs (differentially expressed genes) were mainly enriched in linolenic acid metabolism, starch and sucrose mechanism, phenylpropanoid biosynthesis, hypersensitive response (HR) and systemic acquired resistance. Further analysis suggested that eight candidate genes (*4’OMT2*, *ACLY*, *NCS1*, *GTE10*, *SMO2*, *FLS2*, *SQE1* and *RCOM*) identified by WGCNA (weighted gene co-expression network analysis) may mediate the resistance to agave purple curl disease by participating the biosynthesis of benzylisoquinoline alkaloids, steroid, sterols and flavonoids, and the regulation of plant innate immunity and systemic acquired resistance. After qPCR verification, we found that *AsRCOM*, coding a glycosyltransferase and relevant to the regulation of plant innate immunity and systemic acquired resistance, may be the most critical disease-resistant gene. Finally, the overexpression of *AsRCOM* gene in agave could significantly enhance the resistance to purple curl disease with abundant reactive oxygen species (ROS) accumulations.

**Conclusions:**

Integrative RNA-seq analysis found that HR may be an important pathway affecting the resistance to purple curl leaf disease in agave, and identified glycosyltransferase *AsRCOM* as the crucial gene that could significantly enhance the resistance to purple curl leaf disease in agave, with obvious ROS accumulations.

**Supplementary Information:**

The online version contains supplementary material available at 10.1186/s12864-023-09700-y.

## Background

Growing primarily in semi-arid regions, *Agave sisalana*. hybrid 11648 (*A.* H11648) is a globally cultivated plant known for its tough natural fibers [1]. Besides its use in producing natural fibers, *A.* H11648 could also be utilized for alcoholic beverages [2], nutraceuticals [3], and nanocellulose [4], making it a highly valuable crop. Despite its widely valuable usage, *A.* H11648 also faces a number of challenges due to its susceptibility to various biotic and abiotic stresses, such as cold temperatures [5], root rot disease [6], high temperatures [7], low rainfall [8], low aboveground biomass coverage [9] and agave purple curl leaf disease [10]. Among these various stresses, agave purple curl leaf disease poses a significant threat to the agave industry as it severely damages the plant’s leaves which are the primary source of its natural fibers and will cause a sharp reduction in leaves production by more than 30% in severe cases [11].

Agave purple curl leaf disease is spread through *Dysmicoccus neobrevipes* and has become a significant constraint on the healthy development of agave industry. When *D*. neobrevipes infects the leaves with its piercing-sucking mouthparts, the phytoplasma that it carries enters into the plant, leading to agave purple curl leaf disease and severe damage to plant leaves [10]. Once agave purple curl leaf disease was found on a few plants in one plantation, it almost spread to the whole plantation within approximately two months [12]. Although pesticide application is currently the most effective solution for preventing the disease, it is not sustainable or advisable due to environmental and economic price. In our previous researches, the disease-resistant (*A.* H11648R) plants for agave purple curl leaf disease were found in *A.* H11648 gardens that had suffered from this disease for many consecutive years. Then, planting *A.* H11648R agave is the more prevalent solution. However, the ability of *A.* H11648R agave against purple curl leaf disease weakens over time. Therefore, it is very urgent and critical to elucidate the underlying mechanism of *A.* H11648R agave against purple curl leaf disease and excavate the pivotal disease-resistant genes to cultivate stable disease-resistant cultivars.

Currently, research on plant defense mechanisms against phytoplasma primarily focuses on three areas. The first area involves the external application of plant resistance elicitors, such as benzothiadiazole (BTH) which could significantly reduce the infection of phytoplasma by inducing systemic acquired resistance [13]. The second area is the study of phytoplasma effector proteins, which are secreted by phytoplasma and could affect the growth and development of plants [14, 15]. Lastly, it is research on disease-resistant genes, which enable plants to inhibit the symptoms resulting from phytoplasma infection [16]. Among these three areas, transcriptome sequencing (RNA-seq) has been proved as an effective data mining method and performed to identify a series of crucial phytoplasma effector proteins and resistance genes in a variety of plants. For instance, combined transcriptome and metabolome analysis of *Nerium indicum L*. found that the genes enriched in MAPK-signaling (plant), plant-pathogen interaction, plant-hormone signal transduction, phenylpropanoid and flavonoid biosynthesis, linoleic acid and α-linoleic acid metabolism pathways may play an important role in defensing against the phytoplasma infection [17]. Comparative transcriptome analysis showed that Calmodulin-like (CML) genes and cinnamoyl-CoA reductase-like SNL6 gene may be the key genes against phytoplasma infection in *Indian jujube* ‘Cuimi’ [18]. The *Mu-GsSRK* gene identified by transcriptome and DNA methylome could enhance transgenic plant resistance to the phytoplasma in mulberry [19]. The major latex protein-like 329 gene identified by transcriptome and proteome analysis demonstrated enhanced plant resistance to phytoplasma through altering flavonoid content in mulberry [20]. Until now, the relevant studies on agave purple curl leaf disease are very rare and its resistant mechanism to phytoplasma is still unknown. Therefore, RNA-seq could be an excellent strategy to decipher the underlying mechanism of disease-resistant agave against purple curl leaf disease and helpful for breeding stable and disease-resistant agave cultivars.

In this study, we conducted a transcriptome profile analysis for the disease-susceptible *A.* H11648 and its disease-resistant mutant *A.* H11648R during different processes of purple curl leaf disease. Our analysis revealed that the systemic acquired resistance accompanied with hypersensitive response (HR) may be the primary contributor to increase the resistance to purple curl leaf disease in agave. Moreover, we identified *AsRCOM* as the potential resistant gene by weighted gene co-expression network analysis (WGCNA), and its overexpression in agave could significantly increase the resistance to purple curl leaf disease. This study provides a comprehensive understanding of the resistance to purple curl leaf disease in agave and could be helpful for the development of new disease-resistant and stable agave germplasm.

## Results

### RNA-seq profiles of ***A***. H11648 and ***A***. H11648R plants during different processes of agave purple curl leaf disease

**Fig. 1:**
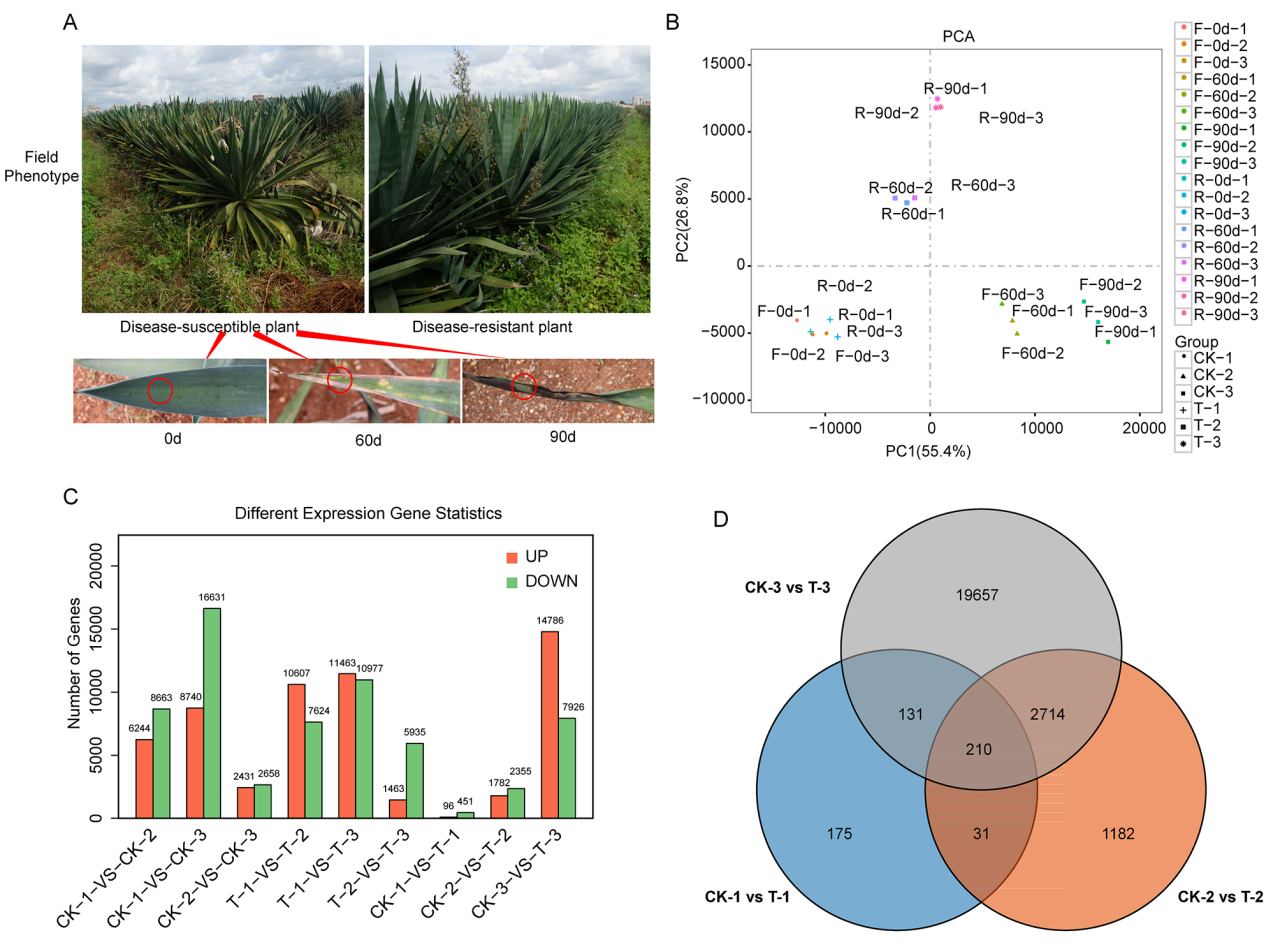
Phenotypes of the *A.* H11648 and *A.* H11648R under the infection of purple curl leaf disease in agave and its RNA-seq results during different processes of purple curl leaf disease in agave. (**A**) The field phenotypes of *A.* H11648 and *A.* H11648R agave. The blade tips of *A.* H11648 and *A.* H11648R were sampled at 0 d (without *D*. neobrevipes, control), 60 d and 90 d after agave purple curl leaf disease infection. The sampled position is marked with red circles. (**B**) The principal components analysis of 18 samples based on the fragments per kilobase of exon per million mapped reads (FPKM). Group ‘CK-1’ indicates three biological replicates (‘F-0d-1’, ‘F-0d-2’, and ‘F-0d-3’) sampled on day 0 in *A*. H11648 agave. Group ‘CK-2’ indicates three biological replicates (‘F-60d-1’, ‘F-60d-2’, and ‘F-60d-3’) sampled on day 60 in *A*. H11648 agave. Group ‘CK-3’ indicates three biological replicates (‘F-90d-1’, ‘F-90d-2’, and ‘F-90d-3’) sampled on day 90 in *A*. H11648 agave. Likewise, group ‘T-1’ (‘R-0d-1’, ‘R-0d-2’, ‘R-0d-3’), group ‘T-2’ (‘R-60d-1’, ‘R-60d-2’, ‘R-60d-3’), and group ‘T-3’ (‘R-90d-1’, ‘R-90d-2’, and ‘R-90d-3’) represent the three biological replicates of *A*. H11648R plants sampled on day 0, day 60, and day 90, respectively. (**C**) The differentially expressed genes (DEGs) statistics among different samples. “CK” represents *A.* H11648 plants and “T” represents *A.* H11648R plants. “1”, “2” and “3” represent samples at 0 d, 60 d and 90 d after agave purple curl leaf disease infection, respectively. (**D**) The venn diagram of DEGs among different comparable groups

In this study, RNA-seq analysis was conducted to better understand the mechanism of agave purple curl leaf disease infection during different stages of the disease. Two agave materials were selected for experimental treatment: *A.* H11648R and *A.* H11648 plants for purple curl leaf disease (Fig. 1A). *A*. H11648 showed yellow blade tips at 60 d, and blackened and died blade tips at 90 d, while *A*. H11648R showed no obvious disease symptoms throughout the infection stages (Fig. 1A). The leaves from three stages in purple curl leaf disease were selected for RNA-seq analysis, which were early (0 day, without inoculation, control), mild (60 day post inoculation, where the blade tips of the *A.* H11648 agave showed obvious yellow spots) and severe (90 day post inoculation, where the blade tips of the *A.* H11648 agave turned black and died, and the neighboring leaf was sampled). Three biological replicates were conducted, and the *A.* H11648R agave showed no symptoms of disease throughout all the *D*. neobrevipes infection stages.

A total of 18 RNA-seq libraries were constructed, comprising of 6 samples which each included 3 replications, resulting in a total of 43,825,614 to 55,003,390 raw reads and 41,657,756 to 52,397,778 high-quality clean reads per library (Table 1). The coverage of mapped reads ranged from 79.22 to 81.64%. The Q30 values, which are an important measure of RNA-seq quality, were all above 95.57% in all 18 libraries. Furthermore, the GC content of clean reads ranged from 48.85 to 51.1%. In summary, our RNA-seq data was high quality and reliable.

**Table 1:** RNA-seq data and quality statistics

Group	Sample	Raw Reads	Clean Reads	Reads mapped	Uniquely mapped	Q30 (%)	GC (%)
CK-1	F-0d-1	46,443,554	44,672,212	35,592,816 (79.68%)	32,858,032 (73.55%)	96.01	49.5
	F-0d-2	53,547,046	51,323,162	41,166,389 (80.21%)	38,037,214 (74.11%)	96.13	49.72
	F-0d-3	53,244,944	50,900,044	40,769,516 (80.1%)	37,597,965 (73.87%)	96	50.43
CK-2	F-60d-1	55,003,390	52,397,778	42,392,273 (80.9%)	39,074,338 (74.57%)	95.81	51.1
	F-60d-2	53,662,486	51,534,668	41,654,526 (80.83%)	38,368,608 (74.45%)	95.73	51.04
	F-60d-3	44,069,444	41,657,756	33,315,257 (79.97%)	30,794,743 (73.92%)	96.08	49.4
CK-3	F-90d-1	43,825,614	42,008,900	33,394,803 (79.49%)	30,691,294 (73.06%)	97.26	50.55
	F-90d-2	44,505,308	42,235,724	33,458,257 (79.22%)	30,837,405 (73.01%)	97.32	49.99
	F-90d-3	44,440,586	42,179,482	33,625,177 (79.72%)	30,882,685 (73.22%)	97.27	50.98
T-1	R-0d-1	54,207,458	51,101,074	40,533,140 (79.32%)	37,377,399 (73.14%)	95.88	50.51
	R-0d-2	48,960,136	46,566,070	37,363,341 (80.24%)	34,517,113 (74.13%)	96.01	50.41
	R-0d-3	51,126,222	48,034,202	38,550,276 (80.26%)	35,535,753 (73.98%)	95.83	51
T-2	R-60d-1	45,701,684	43,500,854	34,724,779 (79.83%)	32,122,366 (73.84%)	96.09	49.23
	R-60d-2	51,962,560	50,173,584	40,098,233 (79.92%)	36,963,972 (73.67%)	96.2	48.85
	R-60d-3	51,055,130	48,607,804	38,985,544 (80.2%)	35,898,290 (73.85%)	95.99	50.39
T-3	R-90d-1	47,490,600	45,701,340	37,311,088 (81.64%)	34,339,621 (75.14%)	95.57	49.42
	R-90d-2	49,831,608	47,201,864	38,430,060 (81.42%)	35,480,538 (75.17%)	95.81	49.69
	R-90d-3	48,235,984	46,319,870	37,798,365 (81.6%)	34,779,277 (75.09%)	96.05	49.65

The rows in the dataset represent distinct samples. ‘F-0d-1’, ‘F-0d-2’, and ‘F-0d-3’ indicate three biological replicates of *A.* H11648 agave sampled on day 0. ‘F-60d-1’, ‘F-60d-2’, and ‘F-60d-3’, as well as ‘F-90d-1’, ‘F-90d-2’, and ‘F-90d-3’, represent the three biological replicates of *A.* H11648 plants sampled on day 60 and day 90, respectively. Likewise, ‘R-0d-1’, ‘R-0d-2’, ‘R-0d-3’, ‘R-60d-1’, ‘R-60d-2’, ‘R-60d-3’, and ‘R-90d-1’, ‘R-90d-2’, and ‘R-90d-3’ represent the three biological replicates of *A.* H11648R plants sampled on day 0, day 60, and day 90, respectively

Furthermore, the principal components analysis (PCA) was performed based on the gene expression data obtained from fragments per kilobase of exon per million fragments mapped reads (FPKM). The results revealed that the 18 samples were divided into five subgroups. CK-1 and T-1 were classified into one subgroup, while CK-2, CK-3, T-2, and T-3 were classified into a distinct subgroup, respectively (Fig. 1B). These findings were consistent with the sample characteristics and indicated that *A.* H11648R and *A.* H11648 agave exhibit diverse transcriptional expression patterns during different stages of agave purple curl leaf disease.

To obtain the accurate differentially expressed genes (DEGs) among different samples (Suppl. Table 1), the cutoff of adjusted p-value < 0.05 was used in this research. A total of 547 (95 up-regulated and 451 down-regulated) DEGs were identified in CK-1-vs-T-1; 4,137 (1,782 up-regulated and 2,355 down-regulated) DEGs were identified in CK-2-vs-T-2; and 22,712 (14,786 up-regulated and 7,926 down-regulated) DEGs were identified in CK-3-vs-T-3 (Fig. 1C). The pairwise group CK-3-vs-T-3 had the greatest number of DEGs out of all three pairwise groups, which also was the time point possessing the most apparent phenotype of purple curl leaf disease in all three time points. In general, a total of 175, 1,182, and 19,657 DEGs were identified in CK-1-vs-T-1, CK-2-vs-T-2, and CK-3-vs-T-3, respectively. Moreover, 210 DEGs were simultaneously identified in all three pairwise groups, and 241, 341 and 2,924 DEGs were identified between CK-1-vs-T-1 and CK-2-vs-T-2, CK-1-vs-T-1 and CK-3-vs-T-3, CK-2-vs-T-2 and CK-3-vs-T-3, respectively (Fig. 1D). The above results suggested that the number of DEGs in CK-vs-T pairwise groups gradually increased along with the extended infection time, which indicated that the underlying molecular immune reactions between *A.* H11648R and *A.* H11648 plants may emerge a significant divergence over time.

### Pathway enrichment analysis of DEGs among different pairwise groups

**Fig. 2:**
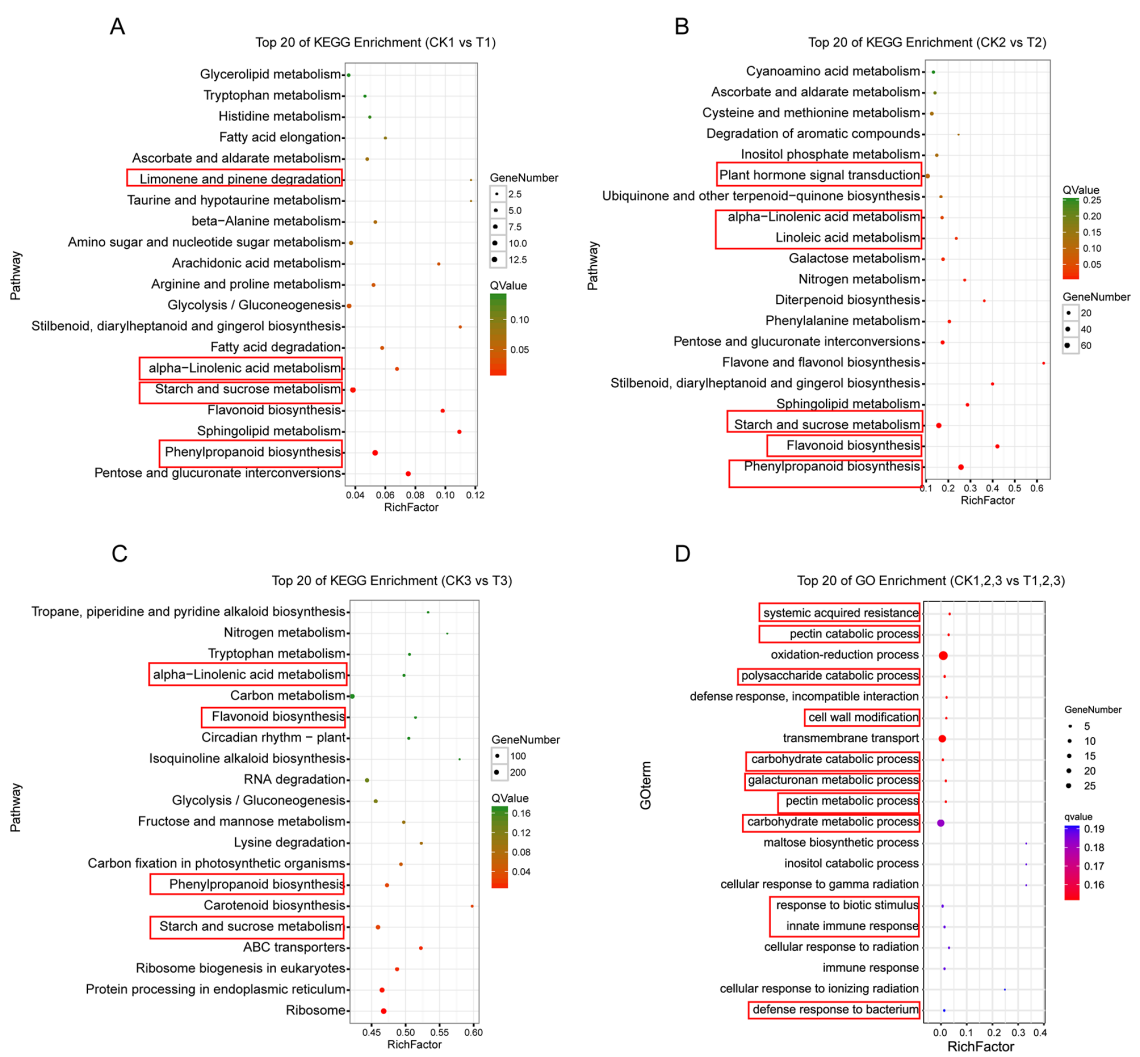
KEGG and GO enrichment analysis for the RNA-seq data during different stages of purple curl leaf disease in agave. The top 20 KEGG enriched pathways are presented for each pairwise group analysis: (**A**) CK-1-vs-T-1, (**B**) CK-2-vs-T-2, and (**C**) CK-3-vs-T-3. (**D**) The GO enriched pathways for the common DEGs among these three comparable groups. “CK” represents *A.* H11648 plants, and “T” represents *A.* H11648R plants. The numbers “1,” “2,” and “3” represent samples taken at 0 d, 60 d and 90 d after agave purple curl leaf disease infection, respectively. The “RichFactor” represents the ratio of the number of DEGs to the total number of agave genes in the pathway. Thus, a higher “RichFactor” indicates a higher degree of enrichment. Qvalue represents the Pvalue corrected by FDR (False Discovery Rate). Enriched GO pathway for the DEGs was defined as significantly enriched pathway when its Qvalue ≤ 0.05. The smaller the Qvalue, the more significant the pathway

To gain a better understanding of the mechanism of the resistance to purple curl leaf disease in agave, we further analyzed the differentially expressed genes (DEGs) among different pairwise groups based on the KEGG and Gene Ontology (GO) databases, expecting to identify the pathways that are significantly relevant to agave disease resistance. In CK1 vs. T1 pairwise group, we found that the DEGs were mainly enriched into the pathways of limonene and pinene degradation, alpha-linolenic acid metabolism, starch and sucrose mechanism, and phenylpropanoid biosynthesis (Fig. 2A). In the CK2 vs. T2 pairwise group, the DEGs were mainly enriched into the pathways of plant hormone signal transduction, alpha-linolenic acid metabolism, starch and sucrose mechanism, flavonoid biosynthesis, and phenylpropanoid biosynthesis (Fig. 2B). In the CK3 vs. T3 pairwise group, the DEGs were mainly enriched into the pathways of alpha-linolenic acid metabolism, flavonoid biosynthesis, phenylpropanoid biosynthesis, and starch and sucrose metabolism (Fig. 2C). Furthermore, GO enrichment analysis for the common DEGs among these three comparable groups were performed. The results showed that the processes of systemic acquired resistance, pectin catabolic process, polysaccharide catabolic process, cell wall modification, carbohydrate catabolic process, galacturonan metabolic process, innate immune response, and defense response to bacterium were significantly enriched (Fig. 2D). Because pectin, polysaccharide, carbohydrate, and galacturonan are all important components of the plant cell wall, we speculated that plant cell wall modification and systemic acquired resistance may play an important role in regulating the resistance to agave purple curl leaf disease.

**Fig. 3:**
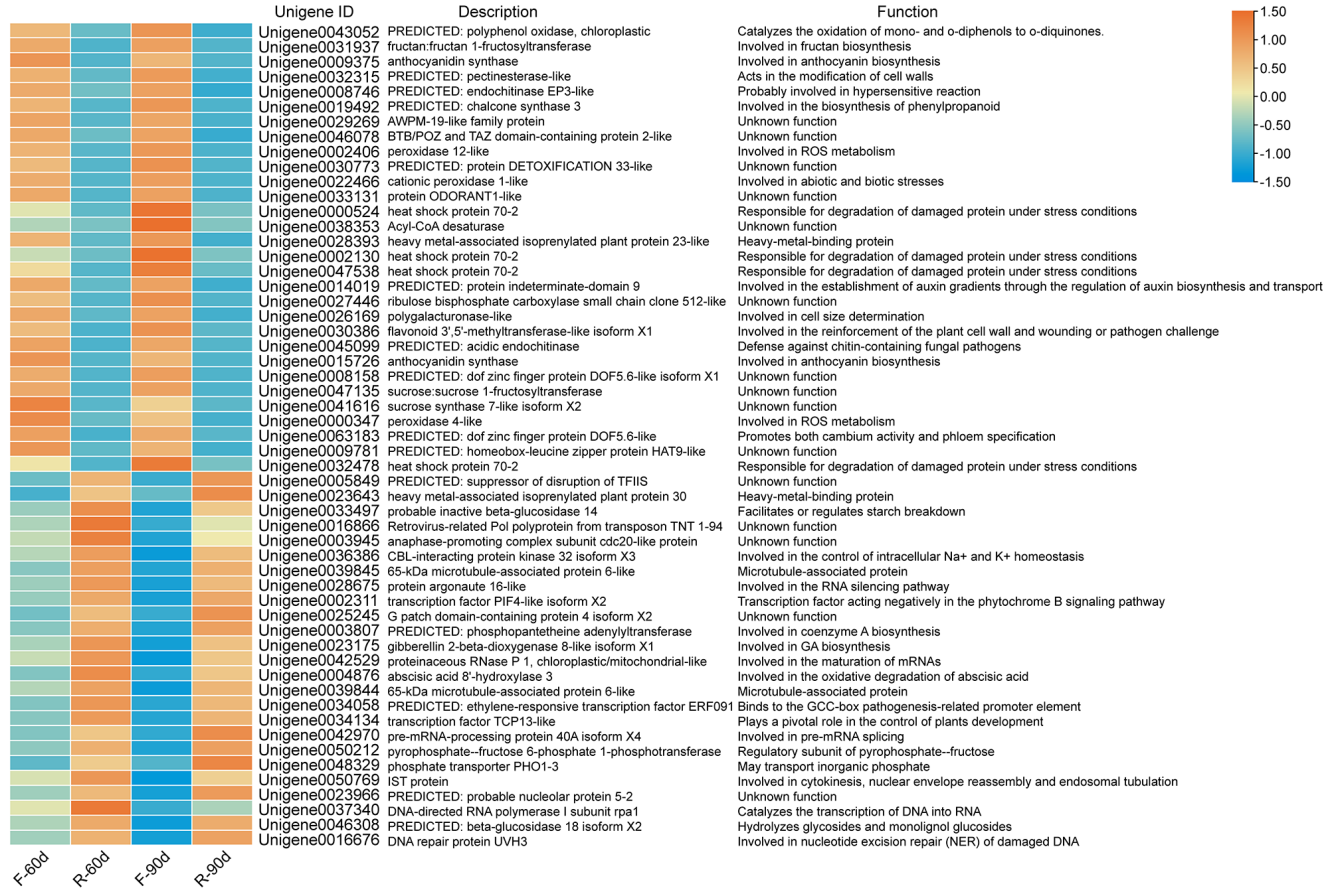
Heatmap for the crucial DEGs enriched by KEGG and GO analysis of all three stages in purple curl leaf disease. “F” represents *A*. H11648 plants, “R” represents *A*. H11648R plants. “60d” and “90d” represent samples taken at 60 d and 90 d after purple curl leaf disease infection in agave, respectively

To strengthen our understanding for the above KEGG and GO enrichment analysis, a comparison between the agave and other species were conducted for the catalogues enriched by KEGG and GO enrichment analysis on phytoplasma resistance in plants. The results suggested that the catalogues enriched in both agave and other species include pentose and glucuronate interconversions, phenylpropanoid biosynthesis, sphingolipid metabolism, starch and sucrose metabolism, flavonoid biosynthesis, alpha-linolenic acid metabolism and other 163 catalogues (Suppl. Table 2). Considering that the number of DEGs at 60 d and 90 d after purple curl leaf disease infection was very high, we chosen 55 representative DEGs at both 60 d and 90 d after purple curl leaf disease infection, which all occupied the top sixty at the absolute value of FPKM (Fragments per Kilobase Million). The heatmap of these representative genes suggested that the down-regulated genes in *A*. H11648R plants were involved in the oxidation of phenols, the biosynthesis of fructan, anthocyanin and phenylpropanoid, the modification of cell walls, hypersensitive reaction, ROS metabolism, abiotic and biotic stresses, the degradation of damaged protein under stress conditions, the establishment of auxin gradients, the reinforcement of the plant cell wall and wounding or pathogen challenge, cell size determination and promoting both cambium activity and phloem specification (Fig. 3). The up-regulated genes in *A*. H11648R plants were involved in heavy-betal-binding protein, starch breakdown, the control of intracellular Na^+^ and K^+^ homeostasis, microtubule-associated protein, RNA silencing pathway, phytochrome B pathway, the biosynthesis of coenzyme A, GA and the maturation of mRNAs, the oxidative degradation of abscisic acid, microtubule-associated protein, development, pre-mRNA splicing, regulatory subunit of pyrophosphate-frutose, inorganic phosphate transportion, cytokinesis, nucleotide excision repair of damaged DNA and hydrolyzing glycosides and monolignol glucosides (Fig. 3). The above results provided a series of candidate genes and its relevant functions which may be involved in the regulation of agave resistance against purple curl leaf disease.

### The important function of hypersensitive response (HR) in regulating the resistance to agave purple curl disease

**Fig. 4:**
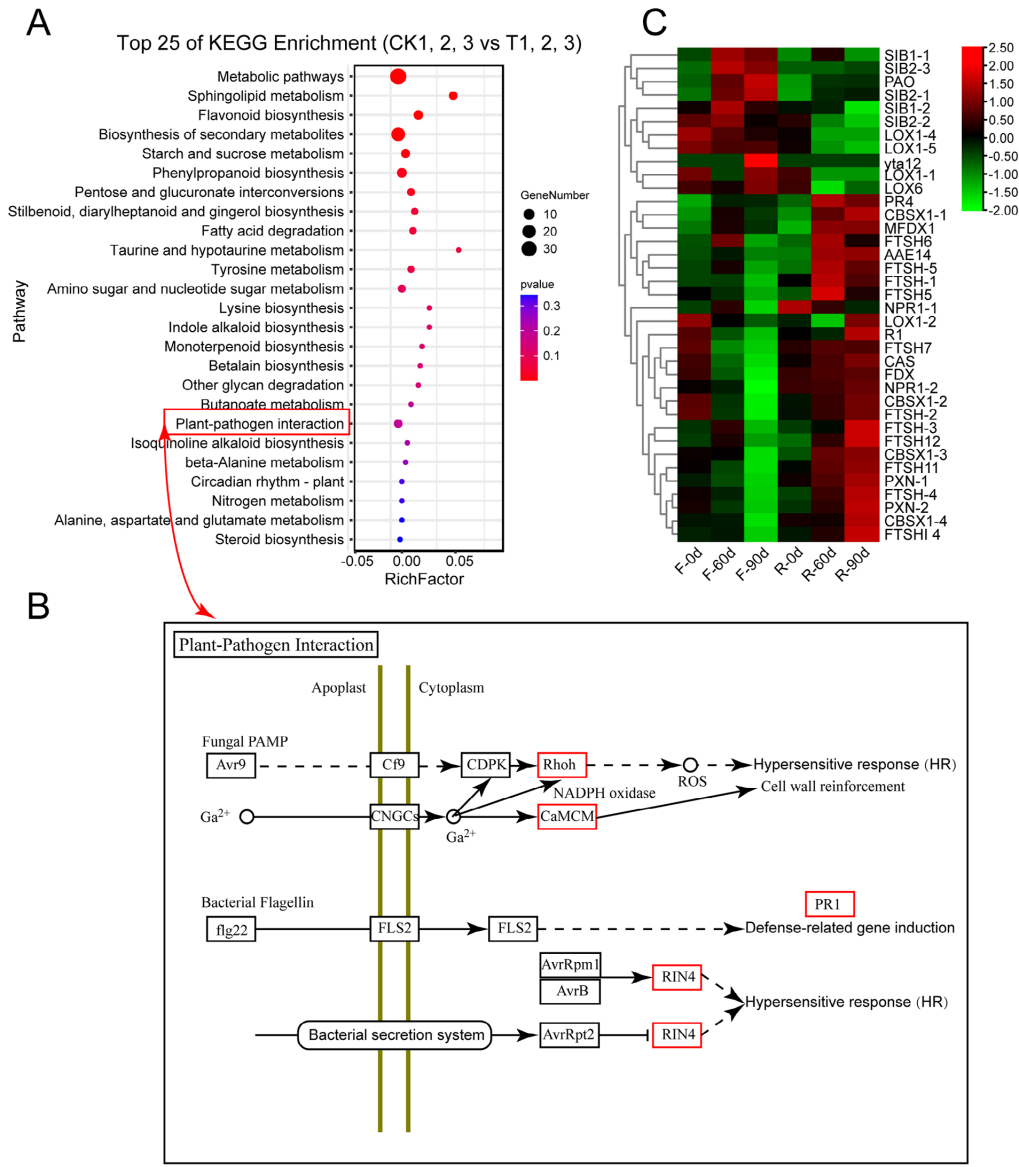
KEGG enrichment analysis of the common DEGs between the *A.* H11648R and *A.* H11648 plants during different stages of purple curl leaf disease. (**A**) The top twenty KEGG enrichment analysis of the common DEGs among the three pairwise groups (CK-1-vs-T-1, CK-2-vs-T-2, and CK-3-vs-T-3) during different stages of purple curl leaf disease. “CK” represents *A.* H11648 agave, and “T” represents *A.* H11648 agave. “1”, “2”, and “3” represent samples taken at 0 d, 60 d, and 90 d after purple curl leaf disease infection, respectively. The “RichFactor” indicates the ratio of DEGs to the total number of agave genes in the pathway. The higher the “RichFactor”, the greater the enrichment degree. The Pvalue represents the enriched degree of the pathway for the DEGs. The smaller the Pvalue, the more significant the pathway. (**B**) The plant-pathogen interaction pathway significantly enriched among the common DEGs between the *A.* H11648R and *A.* H11648 plants during different stages of purple curl leaf disease. (**C**) Heatmap for DEGs related to HR. “F” represents *A.* H11648 plants, “R” represents *A.* H11648R plants. “0d” “60d” and “90d” represent samples taken at 0 d, 60 d and 90 d after purple curl leaf disease infection in agave, respectively

To further clarify the underlying mechanism of the resistance to purple curl leaf disease in agave, we conducted a KEGG enrichment analysis on the common DEGs in the three pairwise groups (CK-1-vs-T-1, CK-2-vs-T-2 and CK-3-vs-T-3). The results of the enrichment analysis were similar to the previous enrichment results (Fig. 4A). Interestingly, the plant-pathogen interaction pathway was significantly enriched, in which pathogen/microbe-associated molecular patterns (PAMP/MAMP) immunity were triggered (Fig. 4B). Besides, Ca^2+^ signal and reactive oxygen species (ROS) burst are triggered, leading to hypersensitive response (HR) and cell wall reinforcement, and bacterial secretion system is also triggered by affecting RIN4 and then results in HR (Fig. 4B). Consistent with these findings, the heatmap analysis of HR-related genes showed that most of these genes exhibited significant expression between *A.* H11648 and *A.* H11648R agave, such as *LOX* (lipoxygenase), *PR4* (wound-induced basic protein) and *NPR1* (pathogenesis-related protein 1) genes (Fig. 4C). Taken together, we speculated that the resistance of *A.* H11648R against purple curl leaf disease may be regulated by HR in agave.

### Weighted gene co-expression network analysis (WGCNA) of DEGs and qPCR analysis

**Fig. 5:**
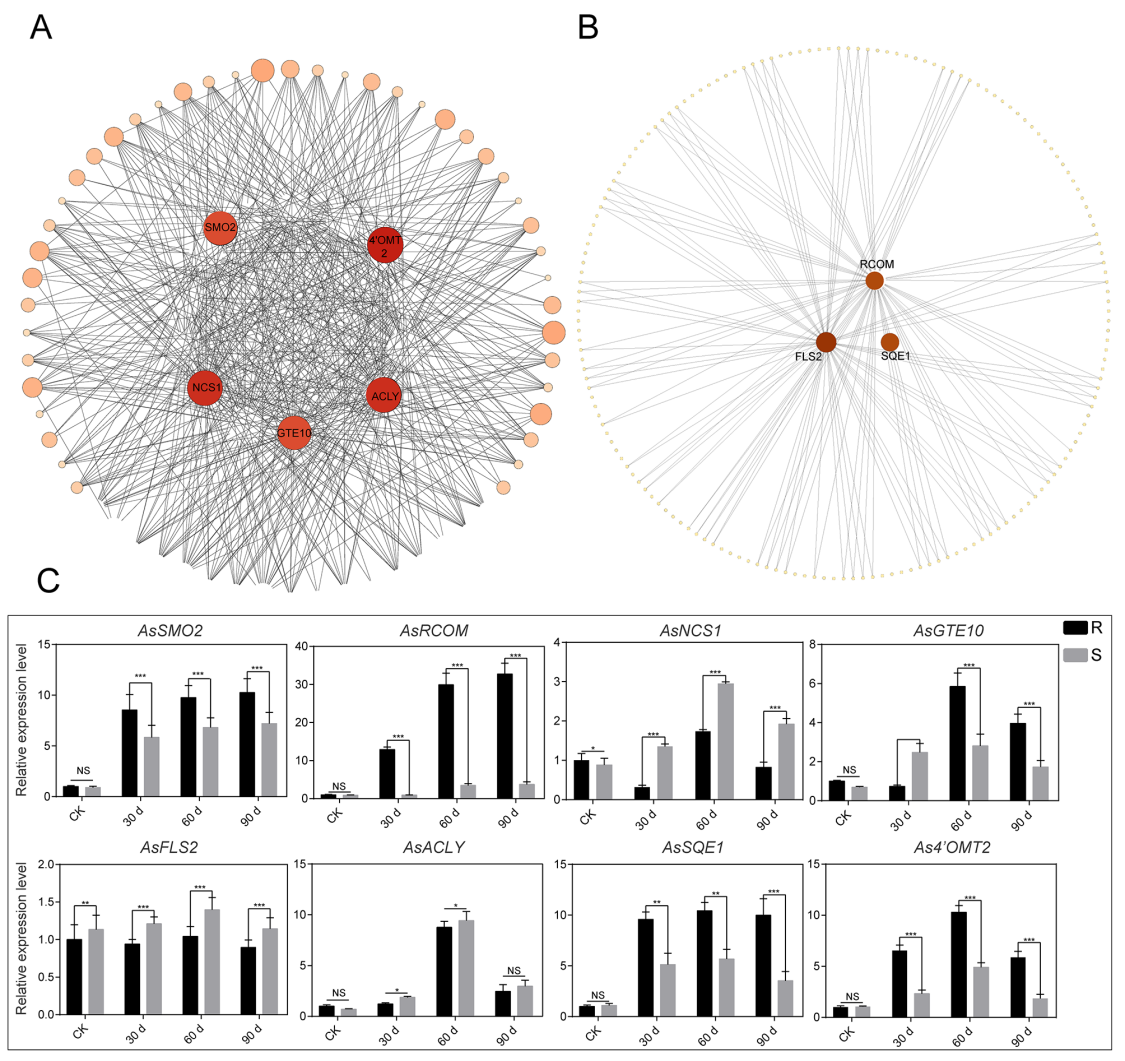
Weighted gene co-expression network analysis of differentially expressed genes (DEGs) and qPCR analysis for the hub genes identified by WGCNA analysis. (**A**) The hub genes identified in the bisque4 module by WGCNA analysis. (**B**) The hub genes identified in the lavenderblush2 module by WGCNA analysis. (**C**) The relative expression levels of hub genes identified by WGCNA at different stages of agave purple curl leaf disease. “R” represents the *A.* H11648R plants, “S” represents the *A.* H11648 plants. Each experiment was performed with three biological replicates. Vertical bars represent ± the standard error of the mean (n = 3, n represents the biological replicates). *P < 0.05, **P < 0.01 and ***P < 0.001 (One-way ANOVA analysis of variance with Dunnett’s multiple comparison test), NS represents no significance

Although we identified 55 representative DEGs in previous section, the vital disease-resistant genes are still unknown. To identify the crucial genes against purple curl disease in *A.* H11648, a weighted gene co-expression network analysis (WGCNA) was conducted. The results showed that the DEGs between *A.* H11648R and *A.* H11648 plants during different processes of purple curl leaf disease were divided into 14 modules based on their expression patterns (Suppl. Table 3). The bisque4 and lavenderblush2 modules were selected to build network analysis maps with Cytoscape software to identify hub genes against purple curl leaf disease, from which the DEGs were mostly up-regulated in *A.* H11648R compared to *A.* H11648 plants. Then, eight hub genes, five in the bisque4 module and three in the lavenderblush2 module, were found to be significantly related to the resistance to purple curl leaf disease in *A.* H11648. In the bisque4 module (Fig. 5A), the hub genes were as follow: *4’OMT2* ((S)-coclaurine N-methyl transferase isoformX1, 4’OMT2) is involved in the biosynthesis of benzylisoquinoline alkaloids and possesses insecticidal and bacteriostatic functions [21, 22]; *ACLY* (ATP-citrate synthase alpha chain protein, ACLY) is involved in the biosynthesis of flavonoids [23]; *NCS1* (S-norcoclaurine synthase1-like, NCS1) is involved in the biosynthesis of the common precursor of all benzylisoquinoline alkaloids [24]; *GTE10* (transcription factor GTE10-like) is a negative regulator of the environmental stress response of the ABA pathway in plants [25]; and *SMO2* (methyl sterol monooxygenase 2-2-like) is involved in sterols biosynthesis and related the metabolism of alkaloids, flavonoids, and sterols [26]. In the lavenderblush2 module (Fig. 5B), the hub genes were as follow: *FLS2* (LRR receptor-like serine/threonine-protein kinase FLS2) is a potent elicitor of the defense response to pathogen-associated molecular patterns (PAMPs) [27]; *SQE1* (squalene epoxidase) is involved in steroid biosynthesis [28]; and *RCOM* (glycosyltransferase family 92 protein RCOM-like) may be responsible for the regulation of plant innate immunity and systemic acquired resistance, the phenylpropanoid metabolism, regulating the resistance to disease in plants [29–32].

To further investigate the function of these hub genes in regulating the resistance to purple curl leaf disease in *A.* H11648, qPCR was performed with the samples from leaves of *A.* H11648R and *A.* H11648 at 0 d, 30 d, 60 d, and 90 d after the infection of purple curl leaf disease. The results revealed that *AsRCOM* gene possess a very high gene expression level along with the whole disease processes in *A.* H11648R compared to *A.* H11648 plants (Fig. 5C). Considering that RCOM may play an important role in the regulation of plant innate immunity and systemic acquired resistance [29–32] and the process of HR may participate the resistance to purple curl leaf disease in agave (Fig. 4), we speculated that *AsRCOM* gene may be the most critical hub gene in regulating the resistance to purple curl leaf disease in agave.

### Functional analysis of ***AsRCOM*** in agave

**Fig. 6:**
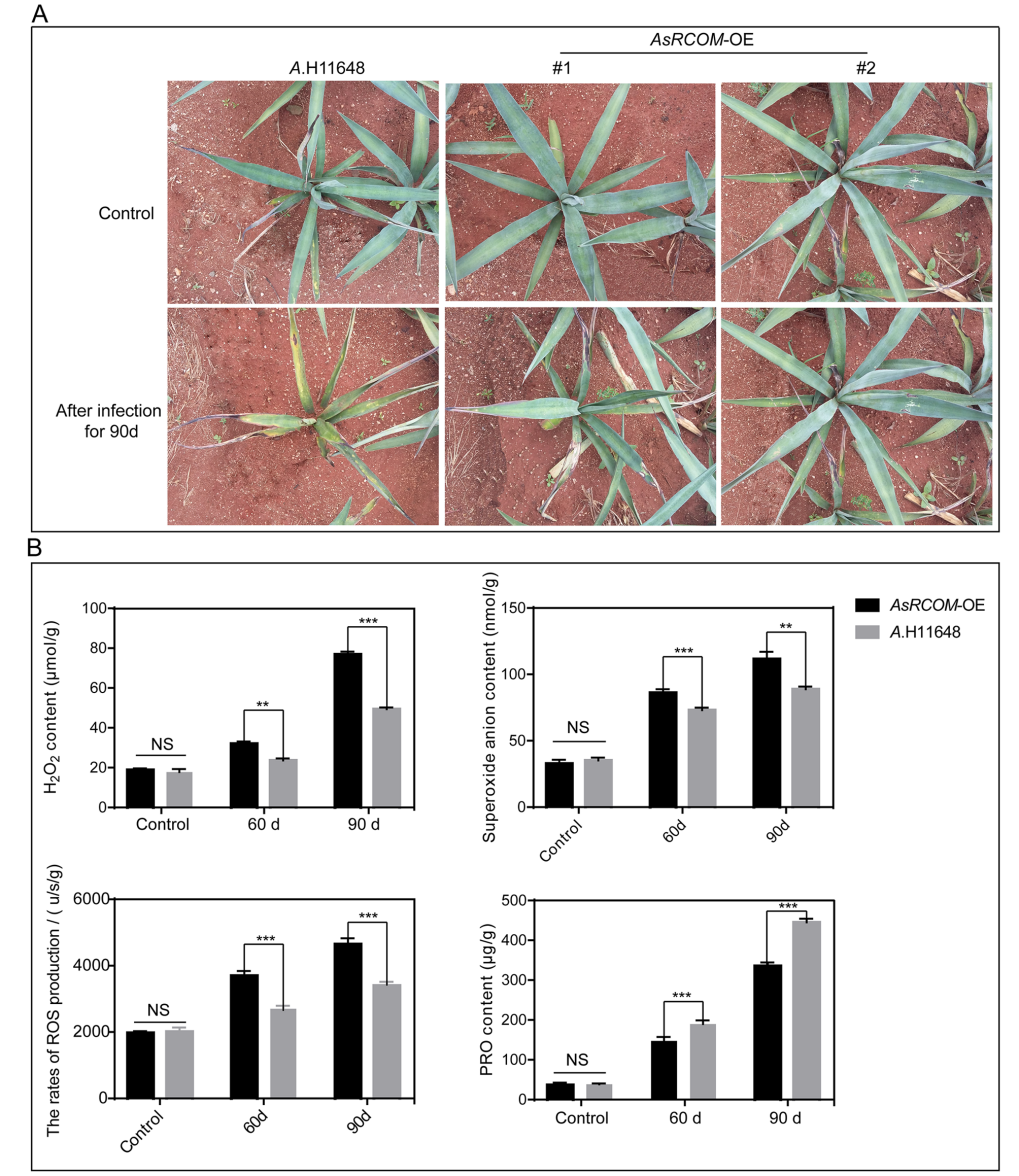
The phenotype and physiological indexes analysis of *AsRCOM*-OE plants exposed to purple curl leaf disease in agave. (**A**) The phenotype of *AsRCOM*-OE plants exposed to purple curl leaf disease in agave. Because purple curl leaf disease in agave was caused by D. neobrevipes, the same number (80) of *D*. neobrevipes were used to trigger the onset of purple curl leaf disease in the *AsRCOM*-OE plants and *A.* H11648. The recipient *A.* H11648 was set as control. (**B**) The physiological indexes analyses were conducted between *AsRCOM*-OE plants and *A.* H11648 during different stages of agave purple curl leaf disease. The leaves of *AsRCOM*-OE plants and *A.* H11648 were sampled to measure H_2_O_2_ contents, superoxide anion contents, rates of ROS production, and proline (PRO) contents at 0 d (control), 60 d, and 90 d after *D*. neobrevipes infection. Three biological replicates were performed for each experiment. The vertical bars represent ± the standard error of the mean (n = 3, where n represents the biological replicates). *P < 0.05, **P < 0.01, and ***P < 0.001 (One-way ANOVA analysis of variance with Dunnett’s multiple comparison test), and NS represents no significance

To confirm our hypothesis that *AsRCOM* is the key gene responsible for the resistance to purple curl leaf disease in agave, we created the *AsRCOM* overexpression (*AsRCOM*-OE) plants in *A.* H11648 mediated by *Agrobacterium tumefaciens* EHA105. The results of qPCR verification for the *AsRCOM*-OE plants found that the expression level of *AsRCOM* genes in *AsRCOM*-OE #1 and *AsRCOM*-OE #2 transgenic lines were significantly higher than that in the recipient *A*.H11648 (Supple. Figure 1), which were selected for the subsequent functional verification. Then, the resistance of *AsRCOM*-OE plants against agave purple curl disease was detected. The results revealed that the resistance of *AsRCOM*-OE plants against purple curl leaf disease was significantly higher than that in *A.* H11648 (Fig. 6A).

Furthermore, physiological index analyses were conducted between *AsRCOM*-OE plants and *A.* H11648 at various stages of purple curl leaf disease in agave (Fig. 6B). The results showed that the H_2_O_2_ contents in *AsRCOM*-OE plants increased by 33.4% and 55.8% at 60 d and 90 d after infected with purple curl leaf disease compared to that in *A.* H11648, respectively. Additionally, the superoxide anion contents in *AsRCOM*-OE plants increased by 18.1% and 25.6% at 60 d and 90 d after infected with purple curl leaf disease compared to that in *A.* H11648, respectively. The rates of ROS production in *AsRCOM*-OE plants also increased by 37% and 36.7% at 60 d and 90 d after infected with purple curl leaf disease compared to that in *A.* H11648, respectively. In contrast, the proline (PRO) contents in *AsRCOM*-OE plants decreased by 28.8% and 32.7% at 60 d and 90 d after infected with purple curl leaf disease compared to that in *A.* H11648, respectively. No significant differences were observed in these indexes at 0d (control) between *AsRCOM*-OE plants and *A.* H11648. These results suggested that the *AsRCOM* gene could significantly enhance the resistance to purple curl leaf disease in agave and the ROS contents in *AsRCOM*-OE plants were obviously higher than that in *A.* H11648 during the different stages of purple curl leaf disease in agave.

## Discussion

Recently, agave purple curl leaf disease has emerged as a major threat to the healthy development of agave industry [11]. Our research has identified a natural mutant from agave H.11,648, which exhibits remarkable resistance to the purple curl leaf disease. However, the underlying mechanism of this natural mutant in increased resistance is still unknown. Elucidating this mechanism and excavating the key resistant genes will be helpful for cultivating stable disease-resistant cultivar in agave. As an effective data mining method, RNA-seq analysis has been used to identify lots of powerfully stress-resistant genes [33, 34].

Therefore, to better understand the underlying mechanism of *A.* H11648R agave in enhancing the resistance to purple curl leaf disease, we conducted an RNA-seq analysis between *A.* H11648R and *A.* H11648 during different stages of purple curl leaf disease (Fig. 1). According to the KEGG and GO enrichment analysis results (Fig. 2), a series of common processes were shared with the RNA-seq results in other species, such as systemic acquired resistance, linoleic acid metabolism, pectin catabolic process and so on. Among these processes, we found that there were twenty representative candidate genes (Fig. 3) were also reported in regulating the resistance against phytoplasma in grapevine [35], mulberry [19], *Paulownia fortunei* [36], tomato [37], apple [38], coconut [39], and Mexican lime tree [40]. And there were thirty-five agave-specific genes that may play an important role in regulating the resistance against purple curl leaf disease in agave, including fructan 1-fructosyltransferase, protein indeterminate-domain 9, flavonoid 3’,5’-methyltransferase-like isoform X1, protein argonaute 16-like, abscisic acid 8’-hydroxylase 3, DNA repair protein UVH3 and so on (Fig. 3). These agave-specific genes are mainly involved in nucleotide excision repair of damaged DNA [41], the oxidative degradation of abscisic acid [42], the RNA silencing pathway [43], the reinforcement of the plant cell wall and wound or pathogen challenge [44], the establishment of auxin gradients [45] and the biosynthesis of fructan [46], which suggested that the processes of DNA repair, ABA and auxin signal pathway, the RNA silencing pathway and the reinforcement of plant cell wall may occupy a vital position in response to phytoplasma infection in agave. Besides, there were thirteen catalogues that were enriched only in agave compared to that in other species, which included non-homologous end-joining, caffeine metabolism, proteasome, glycosphingolipid biosynthesis-ganglio series, glycosphingolipid biosynthesis-globo series, thiamine metabolism, lipoic acid metabolism, glycosylphosphatidylinositol (GPI) -anchor biosynthesis, base excision repair, sulfur relay system, N-Glycan biosynthesis, detoxification, and transcription factor activity, protein binding (Suppl. Table 2). These catalogues exhibited the specificity of agave compared to other species during responding to phytoplasma infection.

Furthermore, as an important tool for excavating the central hub genes in multiple metabolism processes [47–49], WGCNA were conducted in order to decipher the pivotal genes relevant to the resistance to purple curl disease in agave. A total of eight hub genes were identified (Fig. 5), most of which are essential in increasing the resistance to insects and diseases in plants [18–29]. Except for the *GTE10* and *FLS2* genes, almost all of these genes are relevant to the metabolism of alkaloids, a kind of antibacterial compounds [50, 51], which suggest that alkaloids may be an effective external admixture for the defense against agave purple curl leaf disease. The results of qPCR analysis found that the *AsRCOM* gene may be the most crucial gene among all the hub genes identified by WGCNA analysis (Fig. 5C). We also found that the overexpression of *AsRCOM* gene in *A.* H11648 could significantly increase the resistance to agave purple curl leaf disease with large amounts of ROS accumulation compared to the recipient *A.* H11648 (Fig. 6). This finding is consistent with the previous researches that the glycosyltransferase could obviously enhance the plant immunity against biotic stresses [26–29] and ROS accumulation was an important defense responses in plants [52, 53]. Besides, reports showed that enhanced ROS production has been implicated in the hypersensitive response and plant defense signals in plants [54, 55], which further confirmed that HR may play an important role in regulating the resistance to purple curl leaf disease in agave. Similarly, the results of RNA-seq analysis also showed that HR may play a vital role in the interaction between plants and pathogenic bacteria in sugarcane, *Brassica napus*, *Saccharum*, rice, grapevine, *Populus* and sand pear [56–62]. Together, our research showed that the *AsRCOM* gene may regulate the resistance to purple curl leaf disease through HR pathway.

The present study has some limitations. The in-depth molecular mechanisms of *AsRCOM*-OE plants in enhancing the resistance to purple curl leaf disease in agave need to be excavated, including the signal regulation network, the effect of *AsRCOM* gene on the cellular and organic structures, the potential interactive protein or transcription factor of AsRCOM. Subsequently, the problem that whether the *AsRCOM* gene is related to HR should be verified. In addition, the mutants of *AsRCOM* gene mediated by CRISPR/Cas9 system in agave need to be created to supplement the function of *AsRCOM* gene in agave.

## Conclusion

By RNA-seq analysis, we discovered that systemic acquired resistance accompanied with HR may have a crucial function in controlling resistance to purple curl leaf disease in agave. Additionally, we found the AsRCOM protein of glycosyltransferase family identified by WGCNA analysis could substantially enhance the resistance to purple curl leaf disease in agave. Together, our researches provide a profound understanding for the mechanisms of *A.* H11648R in enhancing the resistance to purple curl leaf disease and a series of important genetic resources for breeding disease-resistant cultivar in agave.

## Methods

### Plant materials and samples

Disease-susceptible *A.* H11648 and its natural mutant disease-resistant *A.* H11648R screened from disease-susceptible *A.* H11648 continuously infected with agave purple curl disease for many years were used as experimental materials. Agave purple curl leaf disease was induced by cultured *D*. neobrevipes. During the process of infection with *D*. neobrevipes, *D*. neobrevipes were artificial cultured with fresh agave leaves (Updated every three days) as food sources under the condition of 26 °C, 16 h/8 h light/dark photoperiod. Then, 80 live *D*. neobrevipes were separated from agave leaves by flicking the leaves slowly, and initially inoculated to *A*.H11648 and *A*.H11648R plants all with no *D*. neobrevipes infection, respectively. Subsequently, insect nets were used to exclude *A*.H11648 and *A*.H11648R plants from wild *D*. neobrevipes or other insects until the end of the experiment. Besides, *A*.H11648 and *A*.H11648R plants with no inoculation of *D*. neobrevipes were set as control to confirm that the disease performances were just caused by purple curl leaf disease. Agave blade tips were sampled at 0 d, 60 d, and 90 d post-infection (Fig. 1A), in which the sample at 0 d (On the day of *D*. neobrevipes inoculation, but without inoculation) was chosen as control. Blade tips were sampled for RNA-seq analysis with three biological replicates at each timepoint. All samples were collected and immediately stored at -80 °C for RNA extraction and RNA-seq analysis.

### RNA extraction and Illumina sequencing

Total RNA was extracted from the sampled blade tips using the TRIzol reagent (Aidlab, Beijing, China) according to the manufacturer’s protocol. The concentration of total RNA in each sample was measured using a NanoDrop microvolume spectrophotometer (Thermo Scientific NanoDrop Products, Waltham, MA, USA). RNA samples were treated to remove genomic DNA contamination and cDNA was synthesized using a one-step reverse transcription kit (Transgen, Beijing, China). cDNA library construction and sequencing were completed by Guangzhou Genedenovo, Guangzhou, China. The Illumina HiSeq2500 platform (Illumina, San Diego, CA, America) was used for RNA-seq with 6 GB clean, paired-end data.

### RNA-seq analysis

The quality of the raw RNA-seq data was evaluated using FastQC v0.11.2 [63]. Reads with low quality (N content > 5%, low-quality bases > 30% (quality < 29)) were removed, and higher quality reads were trimmed to eliminate adapters with Trimmomatic (v0.36.5) [64]. The high-quality reads were then assembled using Trinity v2.4.0 (https://github.com/trinityrnaseq/trinityrnaseq/wiki) to generate a unigene library. BlastX (https://blast.ncbi.nlm.nih.gov/Blast.cgi?PROGRAM=blastx&PAGETYPE=BlastSearch&LINKLOC=blasthome) was used with multiple online databases, including Eggnog (http://eggnogdb.embl.de/#/app/emapper), Uniprot (https://www.uniprot.org/), GO (http://wegolgenomics.org.cn/cgi-bin/wego/index.pl), Pfam (http://pfam.xfam.org/), Clusters of Orthologous Groups (COGs, https://www.ncbi.nlm.nih.gov/research/cog), TMhmm, (https://services.healthtech.dtu.dk/service.php?TMHMM-2.0), and KEGG (http://www.genome.jp/kegg/kegg2.html) [65–67], to annotate the assembled unigene transcripts with an E-value cut-off of 10^− 5^. DEGs were identified using the following criteria: FDR ≤ 0.01 and |log2 ratio| ≥ 1 (FDR indicating the false discovery rate and log2 ratio representing log2 of fold change for the RPKM of up/down-regulation). Heatmap analysis of the DEGs in the RNA-seq data was performed using TBtools [68], based on the RPKM (reads per kilobase per million mapped reads). GO enrichment analysis with the identified DEGs was conducted in R-Studio (v3.3.0) with the ggplot2 package (v3.3.5, https://ggplot2.tidyverse.org/). The GO term categories, such as molecular function, were confirmed with a p-value cut-off of < 0.05, hypergeometric distribution, and Bonferroni correction.

### Weighted gene co-expression network analysis (WGCNA) of DEGs

The WGCNA R package (v1.70-3) [69] was utilized to perform a weighted gene co-expression network analysis (WGCNA) using normalized expression values of the DEGs. A range of powers from 1 to 20 were tested for network threshold, and the resulting network was visualized using Cytoscape (v3.9.1, https://cytoscape.org/).

### qPCR verification for the hub genes and its function analysis in agave

In the RNA extraction and real-time quantitative RT-PCR (qRT-PCR) experiments, the TransGen RNA extraction kit (Transgen, Beijing, China) was utilized to extract total RNA from agave leaves following the manufacturer’s instructions. The first cDNA strand was synthesized using the TransGen RT SuperMix (Transgen, Beijing, China) kit. The primers for qRT-PCR experiments and subsequent transgenic detection primers were stored in Suppl. Tables 4, with actin gene serving as an internal reference. The qRT-PCR was conducted using the TransGen SYBR® Green PCR kit (Transgen, Beijing, China) in accordance with the manufacturer’s instructions. Three biological replicates were carried out for each experiment.

The *A*.H11648 was selected as recipient to transform with the pCAMBIA1302-AsRCOM plasmid and pCAMBIA1302 empty vector using *Agrobacterium tumefaciens* strain EHA105. Shoot apex explants were chosen for callus culture in agave. Then the callus were transformed with the relative vectors. After selective cultivation with hygromycin, PCR detection and transplanting to the field, positive transgenic agave seedlings and its recipient *A*.H11648 that all were three months old were planted in the same area covered with insect nets to exclude them from wild D. neobrevipes or other insects until the end of the experiment. Then, 80 live D. neobrevipes were separated from agave leaves by flicking the leaves slowly, and inoculated to *AsRCOM* overexpression and *A*.H11648 plants all with no D. neobrevipes infection, respectively. The phenotype of these plants were observed and recorded to evaluate their resistance against purple curl leaf disease every seven days in agave.

### Physiological and biochemical indexes analysis

The physiological and biochemical indexes analysis was performed as described previously [70]. The H_2_O_2_ contents, ROS production rates, superoxide anion contents, and PRO contents were measured in accordance with the kit instructions (COMIN, Suzhou, China). The leaves of the *AsRCOM*-OE and the recipient agave during different stages of purple curl leaf disease were sampled and performed for the above physiological and biochemical indexes analysis. Three biological replicates were conducted for each experiments.

### Statistical analysis

All data were analyzed using GraphPad Prism 7 software. The statistical comparisons were performed using One-way ANOVA analysis of variance with Dunnett’s multiple comparison test. Differences were considered significant when the *P*-value was less than 0.05. All experiments were conducted with three biological replicates.

### Electronic supplementary material

Below is the link to the electronic supplementary material.

**Supplementary Material 1: Supplementary Table S1**. The RPKM of DEGs between CK-1 and T-1 group

**Supplementary Material 2: Supplementary Table S2**. The catalogues enriched by KEGG or GO enrichment analysis on phytoplasma resistance in agave and other species and the relevant genes in agave

**Supplementary Material 3: Supplementary Table S3**. The modules of RNA-seq data divided by WGCNA

**Supplementary Material 4: Supplementary Table S4**. The primer sequences of relevant genes in qRT-PCR and PCR experiments

**Supplementary Material 5: Supplementary Fig. 1**. The expression level analysis of *AsRCOM* gene in *AsRCOM*-OE transgenic lines by qPCR. A total of three transgenic lines of *AsRCOM*-OE were obtained. The expression level of *AsRCOM* gene in the recipient *A*.H11648 was set as 1. Three biological replicates were performed for each experiment. The vertical bars represent ± the standard error of the mean (n = 3, where n represents the biological replicates). *P < 0.05, **P < 0.01, and ***P < 0.001 (One-way ANOVA analysis of variance with Dunnett’s multiple comparison test), and NS represents no significance

**Supplementary Material 6: Supplement file 1**. The amino acid sequences of RCOM proteins in multiple species. AsRCOM represents the amino acids in *Agave sisalana*. hybrid 11648

## Data Availability

All data and materials are conserved in our laboratory. RNA-seq raw reads have been deposited in the SRA metadata available at https://dataview.ncbi.nlm.nih.gov/object/PRJNA852945?reviewer=fuoka4k1e6knf5434mhljh1uit (BioProject: PRJNA852945), and the RNA-seq mRNA sequences have been deposited in GenBank BankIt submission (Submission ID: 2,723,837; 2,723,846; 2,723,857; 2,723,860).
