# Supplementary material for: A newly identified glycosyltransferase AsRCOM provides resistance to purple curl leaf disease in agave
Source: BMC Genomics. 2023 Nov 7;24:669. doi: 10.1186/s12864-023-09700-y (PMC10629022; doi:10.1186/s12864-023-09700-y)
Supplement: Supplementary file 5 — Supplementary Material 5: Supplementary Fig. 1. The expression level analysis of AsRCOM gene in AsRCOM-OE transgenic lines by qPCR. A total of three transgenic lines of AsRCOM-OE were obtained. The expression level of AsRCOM gene in the recipient A.H11648 was set as 1. Three biological replicates were performed for each experiment. The vertical bars represent ± the standard error of the mean (n = 3, where n represents the biological replicates). *P < 0.05, **P < 0.01, and ***P < 0.001 (One-way ANOVA analysis of variance with Dunnett’s multiple comparison test), and NS represents no significance [file 12864_2023_9700_MOESM5_ESM.docx]

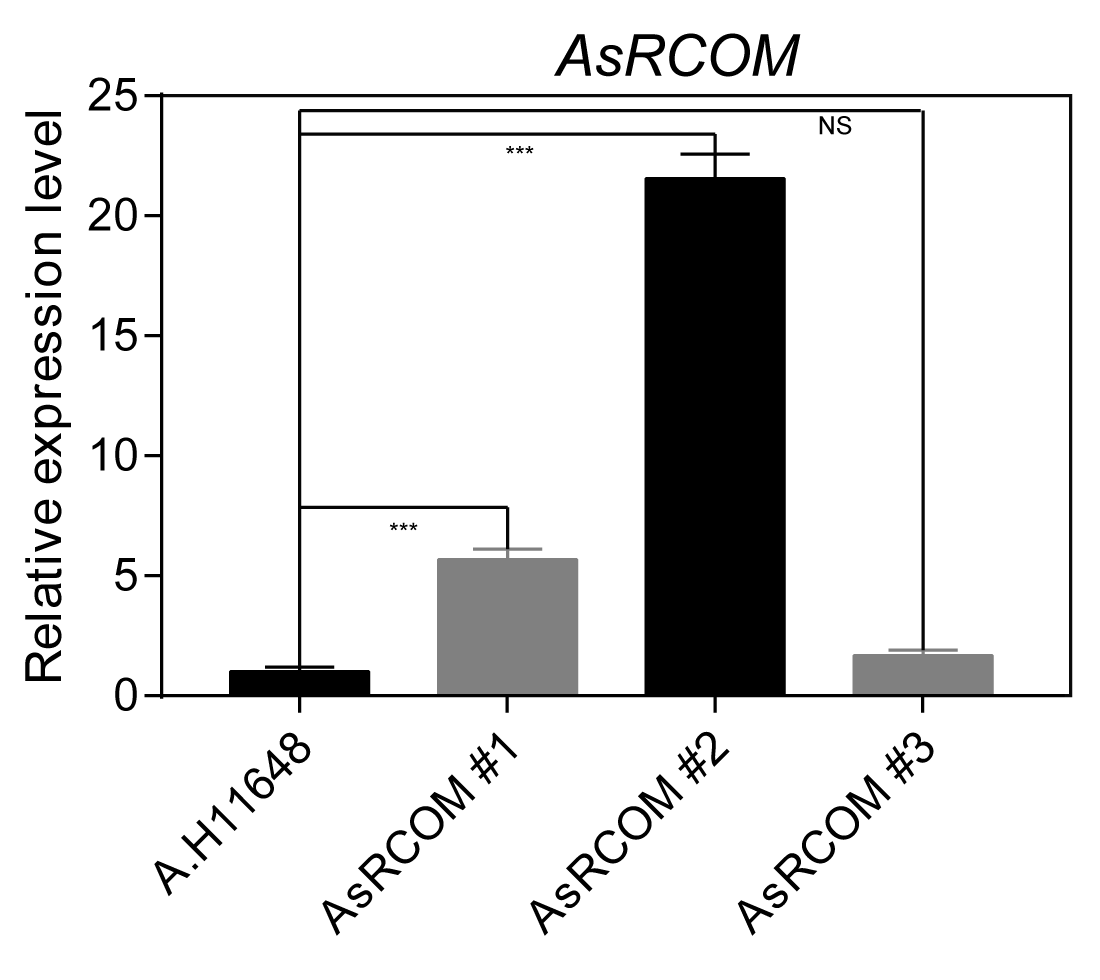


**Suppl. Fig. 1**.The expression level analysis of *AsRCOM* gene in *AsRCOM*-OE transgenic lines by qPCR. A total of three transgenic lines of *AsRCOM*-OE were obtained. The expression level of *AsRCOM* gene in the recipient *A*.H11648 was set as 1. Three biological replicates were performed for each experiment. The vertical bars represent ± the standard error of the mean (n=3, where n represents the biological replicates). *P < 0.05, **P < 0.01, and ***P < 0.001 (One-way ANOVA analysis of variance with Dunnett's multiple comparison test), and NS represents no significance.
