# Supplementary material for: A newly identified glycosyltransferase AsRCOM provides resistance to purple curl leaf disease in agave
Source: BMC Genomics. 2023 Nov 7;24:669. doi: 10.1186/s12864-023-09700-y (PMC10629022; doi:10.1186/s12864-023-09700-y)
Supplement: Supplementary file 6 — Supplementary Material 6: Supplement file 1. The amino acid sequences of RCOM proteins in multiple species. AsRCOM represents the amino acids in Agave sisalana. hybrid 11648 [file 12864_2023_9700_MOESM6_ESM.docx]

>AsRCOM

MRRRLLTLLLPAFLFVLLFANFSHHLIRQSLLSDPPLPDPLTASLIHRPRHQTSHQLALRETKYLLPSPEVTESVLFPDWDVFLLLPSNLTPGDRTNLSCLFSDGATSAANYTGRVPSSARHGFLCKMPRRVQRLRPFYAPRLVGPGLDPAVIGSVQSAKMVRWTRLAYESFSTIDDVIVFAKGVNRYQGKDWPARDVCCVFSVNGAIAAVTPATSSAQEVFRCPHPQLRAAGGGEDVRVTVRIAGEDSPFPSLAAYRHPLPQPRAQDSAEKTKSLMCACTMVYNAAKFLREWVAYHAAVGVERFVLYDNGSDDDLAAALAALAVEGYDVSSVYWPWQKTQEAGFSHCAASQRDACTWMAFIDVDEFLYSPSWALSASPNRSMMVRSVIGSGGERVGQVQVTCLDFAPSGQRAHPRAGVTQGYTCRLRGDARRRRHKSVVRLEAVGPSLRNWVHHFEVGPGWGVKKAGAKEAVVNHYKYQAWSEFRVKFRRRVSAYVADWKERRNEGSKDRARGLGFTEVEPAWWAQGRCEVRDTGLRDAVRSWFAGPTGKLAWE

>RCOM21 Juglans regia; Function: Catalyzes the committed step in the biosynthesis of hydrolysable tannins and biosynthesis of nonstructural phenols (Publication: The walnut (Juglans regia) genome sequence reveals diversity in genes coding for the biosynthesis of non-structural polyphenols)

MRPRVRKALLSCFLAIVLFAFFSVRLSRKAISVATVRYSTSNLTVRSKINPNSLNYAVQENHHDRDAQELSRPRTRHVSSIWDSIATLSVLLPAWEVLVIVPPETPLSPGLGEEYRCIFQNNATSTATFSGVLAFTNRTTFKCVMPDSVRRLRPFLQPILIKQSENESPVTKPMPELIRWTFMAYESFSTEDDVILFVKGVNHRQGLNRSPQEFLCLFGDGTTSFVKTAVTSSIQEVFRCPHPNLMGVISGADSERIKISLEIVTENLVVPSVAYYTPRRSLVIGRPEEPRSILCACTMVHNVAKFLKEWVVYHSMIGVQKFILYNNDSDDDLERVVEELNQDGYNVTTLFWIWPKTQEAGFSHGAVYAKDSCTWMLYVDVDEFVFSPSWDQFKKPSNDMLTSLLSRTPQKTPSSSSLMLPASHRRIGQVVMRCNEFGPSNQTSHPAEGVTQGYTCRRKIEQRHKSMVLLDAMDPSLLNVIHHFQLKEGFRSKTMSMEDAVVNHYKYQAWSEFRTKFRRRVSAYVVDWMQAVNPKSKDRTPGLGFEPIEPKGWAQKFCEVRDERLKLLTQKWFGSTEY

>RCOM Oryza sativa unknown function

MQARRRHARQCRLVVAGLIIVTTLLFFTGDAPRVFIDAPTQNQLPRRLPLSLAAVREAATWPADAVLLPDWEVLLLLHPNATAIAHNATCAFQGAASSPARALGRLPSSGRHAYTCAMPEPARRHQPFHAPRIVAMDAVHASPHDDDELVMMVKWSGRLVYDSVVVDGGDVLVFAKGVNPRQGVNRPASDVRCVYYRGRGGSADDVVASLPAATSAQQVFRCPPPPPAALLRVTLALAGEEEPIPSVATYSLPPASAAATHKRRHKICACTMVRDVGKFVREWVAYHAAVGVGRFILYDNGSEDDLDEQVRRLTAEGMDVTTLAWPWPKTQEAGFSHSAAVHRDACEWMAFIDVDEFIFSPNWATAASPSSSMLRSIVAVKPDVGQVSLGCVDFGPSGRTTHPPEGVTQGYTCRRRAVERHKSLLRLEAAERSLVNSVHHFELREGKRGEWNRRARVNHYKFQAWDEFRLKFRRRVSAYVADWTHRVNLQSKDRTPGLGFDPVQPAGWAAKFCEVNDTLLRDVTRRWFAAAGESQLQAAR

>RCOM1 Musa acuminata subsp unknown function

METSMVCLPGPSSSPSDSVSATMRRKLTTSLLSIGVFVLLAASFSFHVSRDFLPPAALNPATLTQPIDRLMNHAVHELPASLRRLQSPLPMDSVLLPDWEVLLLLPLNSSSAAAGGAKLFCLFHTGATSPALPAGPSSFRCSLPNSVRRVRPFYTPRLSGATSAAAPSRGQEDPPREMIRWSTRLTYESLSTAHDVIVFAKSVNHRQGIGRPAAGLRCVFSPVSGGGPVAWTIATSSAQEVFRCPHPPAADLSSAVPMRVSLATEPEAAPIPTVASYRTPRVQETDSKALPGRARVCACTMVYNVAKFLPEWVAYHAGVGVGRFFLYDNGSEDELDAAVSRLGSEGFNVTTRYWPWPKTQEAGLSHCAAANRDACEWMAFLDVDEFVFSPAWADSDRPDRSMMGSLLAVEPEVGQVSIRCLEFGPSGHRAHPRLGVTQGYTCRRRKEQRHKSVVRLDAVAHSLVNSVHHFRLREGFRTRWAAAGQARVNHYKYQAWDEFKAKFRRRVSTYVADWKETTNLGSRDRAPGLGSEPIEPRGWAGMFCDVNDTLVRDATHKWFSAAGPGGAHRMVWQH

>RCOM2 Tripterygium wilfordii unknown function

MATPKKPLSSPPGLAKGNFVLLVETMSRRANSKLFVFAFLSLLAFLSVSFYGSRDVNYQTDLDFPPTRPKITNPSSHAITITENVDEELSRGLNRRVSSVKDSSVDSVSVLLPDWEVLMIVSPRSVVDSGESLWCLYANNATSKAIFAGVLPSTNQTTFKCLFPESARRRKMFLQSVLLTRSPEKEKSSEAAPMMIQWSFMAYESFSTEDDVVLFAKGMNNRQGINRSPSEFNCVFGNNGAKTAVTSSTQEVFRCPHPNVTAFDSGGEDDDRHKIKISLEISGENLLVPSVAYYNPPLTIALPRRKSLLCACTMVYNVAKFLREWITYHSKIGVDRFILYDNNSDDDLQTIVKDLNQEGYDIETLLWIWPKTQEAGFSHGAVYANDSCSWTMYVDVDEFIFGPNWNNSENPSDQMLKSLLPSSSERHHHHQIGQVSLSCNEFGPSDQKQHPVEGVTQGYTCRRLEENRHKSIVLLDAIDHSLLNVIHHFALKENYRSKQVSMEVALVNHYKYQAWPEFKSKFRRRVSAYVVDWTKATNPKSKDRTPGLGFEAIEPQGWAHKFCEVRDERLKNLTQTWFGNKTSNGLKMAWQR

>RCOM3 Dioscorea cayenensis subsp unknown function

MMKLHRLPALLFVLIVSTILIVSINLRRSFSFPGAVSGPVTDFSIPLPRRHTQSLALLDHPLPLRLHSQPSIDAVLFPDWETLILLPLGFPPPSTPLSCHFPNGVISPAVLSGVIPSSGRQTLRCSIPPSARRRLSPPQIPGTAPPSELLRWNKLVYESISIPDDVIVFAKGINNRRQGVNLPATELRCVFLSIDGAVIAFTPVTSSVQEVFRCPHPNLNPSSGDALRVSLQRNRDPRAFPSLATFKPFSRGPHPSAAQDKTRPLLVCACTMVRDVSKFLGEWVTYHSAIGVDRFIIYDNGSEDDLGSAINRLLLEGHDVSTVLWPWAKTQEAGFSHCAAAHRDSCDWMAFIDVDEFLFSPAWAESARPNRTMVGSILPTEPESDRVGQVSVPCRDFGPSGRKEHPRNGVTQGYTCRARAQERHKSVVFLQAVARSMVNVVHHFGLGEGWRTASVGSKRAVVNHYKFQAWPEFRTKFRRRVSAYVADWRNEINMNSKDRTPGLGSEPVEPAGWERRFCEVNDTRLTELNRKWFGLDQGRMVWE

>RCOM4 Phoenix dactylifera unknown function

MRRGFTTSLLLIAIFVALIRSYVHLFGDLLLPYATLDGDDAGAPPPPPAAFPLNANLTQPLSHRPNFAVRELLRHPSARLSPPPPESILFPDWETLLVLPLHSAAIASAGNHRNLSCLFHNGAVSPAGFVGTLPTSPARAAFRCPIPSSIRRLRAFYTPRLIDPAAAAAAPESDGHDPQSEMIRWTRLAYEAVSTPTDIVVFAKGVNTRLGVNRPPSDLRCLFSSYASGDDAPVAATPAASSAQEVFRCPHPAATAIAAKSRTGEALRVSLEIAAEGIQIPSLANYPWPPVARRTTEGRALICACTMVRNVAKFLAEWIVYHSAIGVERFFLYDNGSDDGLHSVVGRLASDGFNISTRFWPWPKTQEAGFSHCATVNRDTCTWMAFIDVDEFIFSPAWAGSELPNRTMLGSLVAVRRNVGQVSMRCFDFGPSGLRAHPRDGVTQGYTCRRRKLERHKSLVRLDAVDDSLVNSVHHFELKTGFGTRRTGVSAARVYHYKYQAWEEFKVKFRRRVSAYTVDWTETVKLRSKDRTPGLGFEPVEPKGWANRFCEVKDSRLRDVNRKWFRVEGPNEDFRMAWEWDGRRERTRERERDSFS

>RCOM5 Rhynchospora pubera unknown function

MVKIKNLSIILSVISLFCFLFSYYILLSSTGTIKPEINQSRQHKSIHAVQESHQPIDRHHIITSHQPYAILLPDWEVLILLPPHAFSAYAHSKMMCQFQNNVISAAFFSGIIPSSGRSSFRCILPKSLHRIRPFLSPRLISTSITPDSSSNGSQEPQFDCYSAPEMMRWTRLVYDAVTTPQDVIIFAKGLNKRSGTNLPVSDVECVYLSVADGRAATYAAINAAQEVFRCPHPTSKDFNILNSSSVVRVSLAVATNKTPIPTLATYSPPRAPVKNTKMTNQSLICACTMVFNVAKFLREWVIYHASIGVEKFFLYDNGSEDELGPTVSQLLVEGYDVSSYYWPWPKTQEAGFSHCAAVNEKTCQWMAFIDVDEFVFSPAWAASHQPNHSMLASLVEPIGRKVGQIMIYCNEFGPSGHRTQPKNGVTQGYTCRRRSKERHKSIVQLRAVDPSLVNSVHHFKIKDGFETKRLPSVQINHYKYQVWEEFRMKFRRRVSTYVVDWKDKLNLNSKDRAPGLGVEPLEPDGWAYKFCEENDTLLRDVTRQWFGTEESDRRFKMKWERT

>RCOM6 Punica granatum unknown function

MTSSFSSSSVIITISPSPSLNSLLAYEAAMVRRKVRTTFFLISTALFAFFYLLLHLRLSPFLAANASSSSSSSSSALDIDTDLGQGLRHLNLPRSFHDSADLQHFVVSEPEPNRTRVTSSISEDAAVEEPKAEEPKVEEPVAILFPDWEVFVVADPSSVSPGDELHCVFQNNATSPARFAGVLPFTNRTTFRCLLPPSVRRLRPFYQPLLTSFPRGPHPVRSHSPPPELFRWNFLAYESFSTEDDVVLFVKGVNNRQGINRPPSEFSCVFFNGEDPSAAVKTPVTSSIQEVFRCPHPKLRSPAEGVDGEKLAFKISLEIAREKLVVPSVAYYAPHRASSPRASKSLICACTMVYNVAKFLREWVTYHARVGVERFVLYDNGSEDDLAGAVEELNKDGYDVHTIMWPWPKTQEAGFSHSAVYHNDSCSWMMYVDVDEFVYVPSWDSQEQPSNKLIESSLLPQDSHGIRSRVIGQVSINCNEFGPSGQRSNPPDGVTQGYTCRRRVEQRHKSIVRLSAVDLSLLNVIHHFKLRQGYLSKQMGLNRALVNHYKYQAWPEFQTKFRRRVSAYVADWTQAVNPMSKDRTPGLGFEPIEPKDWADKFCEVRDKRLKLLIQKWFGAETESGYRMPW

>RCOM7 Magnolia sinica unknown function

MPEKRKKMRIIRTAAPLFFVIISAFLVASFYHNVFHVHLRHLSPSSLSPSKNQNPNLAIREDLIRGPDPSPLRAPPDSILFHDWEVLVVLPENPFPSSASALGHTCLFQDGAASPALPAGVLPRGARATFKCRMPFSVRRLRPFFTPALTRTPEILRATPAFVGNSPEMLRWSSLAYESISTEDDVIVFAKGVNGRQGVNVPPSALRCIFGNGVTTAVTSSVQEVFRCGKPDRPIPGDEVKITLEIRTESVDRAHPSVALYAPRQRTLAAEAEPALICACTMMYNSGKFLREWVMYHATIGVERFVLYDNGSDDDMADVVEKLLEAGYHVTTILWPWPKTQEAGFSHCAVSYRDTCKWMAFVDVDEFIFSPSWTNASQPSKHMLESFLPKSPSSSSSPSPSIGQIALKCFEFGPSNRQSHPKEGVTQGYTCRRRIEQRHKSIVLLDAIDSSLVNVVHHFKLKDGYRTLRLSREVAAVNHYKYQAWLEFKKKFRRRVSAYVVDWRNPTNLASKDRTPGLGNNPIEPVGWAGKFCEVNDSRLRDVNRQWFGLESPTGYKLAWEDG

>RCOM8 Quillaja saponaria unknown function

MTTIIKIPNSPPPSPPPTRNSLVFSNSIHSPNNHQNPSSCLSVILSFLETMRRRPRATFLITALTILLFAAFSLHLSRNAISGDDLRWYPGSNFTTPSKHNDRVNYVVRENVDPVEDLSHRSRRVTSIRDSIDTVSVLIPDWEILVIVSLDTPLSPNPADQCRCLFQNNATSPARFSGVLPFTNQTTFKCVMPDSVKRRRPFFQPILTKSAENEPRSPGPELLRWTFLAYESFSTEDDVVLFVKGVNHRQGINMPPQDFRCVFGYGTDDAVKTPVTSSIQEVFRCQHPKLSGGNEKMKMSLEIIGDNLVVPSVAYYTGRRTIANSKPKSLVCACTMVHNVGKFLREWVMYHSKIGVEKFILYDNNSDDDLESVIKKLDGEGYDVSSVFWIWPKTQESGFSHSALYAKDWCTWMTYVDVDEFIFSPLWQNSTHPSNLMLHSLLPPTPQQLIGQVSIHCNEFGPSGQRSHPIQGVTQGYTCRRRVEQRHKSMVLLDAVEESLLNVVHHFKVKENFMSKQISLEEGVVNHYKYQAWPEFRRKFRRRVSAYVVDWTEAVNPMSKDRTPGLGFEPVEPEGWADKFCEVVDERLKSMTERWFGSLTRNGYTLAWQR

>RCOM9 Malania oleifera unknown function

MRRKVRTTLSLIFAAALLLAVASLNGFNRAISSDNHPVSSLSFASGRSNNNNNPSHKLSYAIREDFSSRQSLRHVSSVVAESIAADSVLVPDWQVLVVVSPDSVLFPDDEYSCVFQNNDSSPARPAGVLPFTNRTTFLCELPNSVRRLRPFFQPALTRASAKAAENLTPSRELIRWNFLAYESFSTANDVVLFAKGVNNRQGINRSPAELSCVFSSDSGVTVRTPVTTTAQEVFRCLHPPDDAIPISRDDRITISIEIVEENRILPSLAHYAPGRNLAIRKEKSLFCACTMVYNVAKFLKEWVKYHSSIGIDKFILYDNGSDDDLQKVIDELQEEGYNTRSLLWPWSKTQEAGFSHCALLAKDSCTWMIFIDVDEFIFSPSWLDSSQPSKLMLKSLLPKNNSITASSISSSSSPSVSSLRPVGEISLYCYDFGPSGQRSHPVEGVTQGYTCRRRAEERHKSMVLLEAVDESLLNVIHHFRVKQGYRVKKVRKEVAVVNHYKYQAWPEFKAKFRRRVSAYVVDWRQEVNPASKDRTPGLGFRPVEPAGWADRFCEVKDEGLKILTQRWFGVPRESWYEMPWQ

>RCOM10 Cinnamomum micranthum unknown function

MRARIHTAVITVFVSVVLVASISHHLFHDIFTIDIISLSHSYLSKPTNPNLSILDQPPSSPSKSLYPNNITSQSPSNSPSPSPSTPSSHTKPGCGAHLLPARDPPDSILFPDWEVVVVPPLSNLPHPDPTQPSTCLFQDRNNSPADPGGVLPFGSRAIYRCVMPLSSRRQRPFLSPALTDSPEKADICQNQPHMIRWNFVTYESLSTENDVILFTKGVNSRQGVNLPAANLRCKFADGVTTAVTHSFQEVFRCPHPARPVPDLTRITLEIGPEDGNTRVVPSVAAYEADRRTLAGGDAGRSLLCACTVVYNVAKFLKEWVMYHSRIGVEKFVLYDNGSDDDITNVVAHLARSGYDVTTFLWPWPKTQEAGFSHCATRMNDTCTWMMYVDVDEFVFSPSWQNASQPSKYMLQSLLPNDTITSSSLKIGQVTISCLEFGPSNRRSHPKEGVTQGYTCRTRKEQRHKSIVRLDAVDLSLLNVVHHFDLKVGYKWKKVPLEQVVVNHYKYQAWSEFKNKFRRRVSAYVYDWRDGLNPKSKDRAPGLGSKAIEPEGWAQRFCEVNDTQLRDVGRSWFWLKEPSGDRLAWQDE

>RCOM11 Panicum hallii unknown function

MQSPPSPRLLLLLALALAALLAFLTSTPTAALHHYAASSSSPARALLVPQQPRTQHRLTLRAVREDASSATPANDDDRYPLQDAVLLPDWEVLVLLHPAAPESSSNATCAFPGGAASPARSLGRMPASGRQAYTCAMPRPERRRNKPFRAPRLVTTTTAPSSQSQSQQSRWPRPEMLLRWSGRLAYDAVALPGTGDVLVLAKGVNPRQGVNRPASDVQCVYYRHNATGDGVVASLPAATSAQQVFRCPAPPATAGDLRVTLAVAGGEPIPSMATYSPPTAASGGGSSSAHNKNSKKVVMCACTMVRDVAKFLREWVVYHAAVGVDRFLIYDNGSQDDLEGEVRQLSAAGFDVSTHVWPWPKTQEAGFSYAAAAHRDSCEWMAFVDVDEFIFSPRWAESSRPSKSSMLRSVVAAVEPDVGQVSLGCKDFGPSGQTKHPEEGVTQGYACRRRAEERHKSVVRLDALEPSLMNSIHHFEVRPEFRWERSRQARVNHYKYQAWDEFKVKFRRRVSTYVADWTDPVNHGSKDRTPGLGFEAVEPEGWAHRFCEVEDTLLRDATRRWFGVGFTSRPS

>RCOM12 Rhodamnia argentea unknown function

MPHSKPLPPLESITMPSKVRTTFLLIAVALLAFFHFHLRLSPSGAGAGDLRFGLSNLSVARPSEQSSAVVALNYAIHEAESVPSRHVSSIPEEPIDTVSILFPAWEVFVIAPPAEADDRVREGDWHCVFQNNATSPAALAGVLPFTNRTMFKCELPPSVRRLRPFYQPALTRSSSERAIPAIATEGVAEELLRWNFLAYESLSTEDDVVLFVKGVNNRQGINRSPSELSCVFGDDPTTAVRTVVTSSAQEVFRCAHPKLSAVNSGTEIKIKITLEVAVENRLIPSVAYYTPERLRLARNGSRSLLCACTMVHNVAKFLKEWVMYHASIGVDRFLLYDNGSDDGLQSVVDDLNRGGYDVRMLTWPWPKTQEAGFSHGAIYANDSCEWMAYVDVDEFVFAPAWNSSALPSKLMLTSLLPAQDGPALHGQVSIKCYEFGPSHRKTHPPEGVTQGYTCRRRIDQRHKSVLRLDAAHASLLNVIHHFRLKEGYKSKQVRLEDGVVNHYKYQAWSEFKTKFRRRVSAYVVDWTQATNPASKDRTPGLGFEPVEPSGWAERFCEVRDYTLKALTRRWFGRESGSGDTMAWQR

>RCOM13 Lotus japonicus unknown function

MLSIFNQKFIQTLFTCNRPRLTMRRRTTFLLSLLAILLFATFSLHLSRNAISTSQPYSYLHNANFNTNENVAAVRDIVNHQTRRVSSVKASPSTTVSVLLPDWEILVLVSPNTHSSSSPDDHRYCLFPNNARSPASYSGVLPFTNRTTFKCDLPESVRRRRVFPQPMLVSGTPETESPVSSPAPELMRWNFLVYESFSTDDDVVVFAKGVNHRQGYDRSLNELRCVFKLADGGDSINTAVTSSAQEVFRCAHPDLDLNSDHALSENGNRIRISISLEIIGENLVIPSIAYYRPRPSREAQAQAQPKHFLCACTMVYNVAKFLREWVMYHTKVGVENFILYDNGSDDDFAGEVKNLRSEGYNITTLLWIWPKTQEAGFSHSVVYSKAKGLCNWIMYVDVDEFVYSPAWRIGDESPISSPSLKSMLVGSEDENGNGIRVGQVSMRCLEFGPSGQRLHPAEGVTRGYTCRRKVERRHKSIVLVEAVDRGLRNVVHHFEVKEGLKWKQVRVEEMLVNHYKYQAWDEFKSKFRRRVSAYVVDWKKNVNLGSKDRTPGLGFEAVEPEDWANKFCEVRDERLKLLTQAWFGSYNNASSRL

>RCOM14 Setaria viridis unknown function

MASSPKRRPSRPLCLLSPLGKKAQHYASSVSSSSPARALLVPPPRTSSSSSNSKRLTLRAVREATSTPAYHGDPPDAVLLPDWEVLVLLRPGAPAPPGGNATCAFPGGAASPARSLGRMPASGRRAYTCDMPRPERRHKPFRAPRLIATSMTSSDDQSSESQSTPPLMLLRWSGRLVYDAVALHSTGDVLVLAKGVNPRQGVNRNASDVRCVYYRDNNNATGSVVASLPASTSAQQVFRCPPPPTTTTDDLRVTLAVADGEPIPSMATYSPPSPTPTDEKNKKVAVCACTMVRDVAKFLREWVAYHAAVGVDRFFLYDNGSQDDLEGQVRQLNSAGFHVSTHLWPWPKTQEAGFSYAAAVHRDSCEWMAFVDVDEFIFSPSWAPSSKPTKSMLRSIVAAVEPDVGQVTLGCKDFGPSGQTKHPEEGVTQGYTCRRRAEERHKSLVRLDAVDPSLINSIHHFELRPELRWERSQQARVNHYKYQAWDEFKVKFRRRVSTYVADWTDPVNHGSKDRTPGLGFEAVEPAGWAHKFCDVEDTLLRDVTRGWFGVGFSSNKLRRLGPPTHYSSS

>RCOM15 Nelumbo nucifera unknown function

MRRRIHTPPALSIFISVLLLASFSLYLSRDVFSGEESRPSPSLFRKRFSNLPLNFAVTENQYRHRSSHHVSSLGELSPDAILFPDWEVLLVLSPRDSLPSDSSDGYFCLFQNNATSPARPTGFLPFHQLVTFKCVLPSSVRRLRPFYQPILTKSPEYYWLGNDSGSPELLRWSHLAYESLSTENDVVLFAKGVNQRRDRSRPPSELRCVFGDDATKAVRTEVTISSQEVFRCRHPDEASLRRLFNGRDDERIKISLEVRQQKKVTVVPSLAYYSGPRKVTSEKGKSLVCACTMVYNVAKFLKEWVIYHSKIGVDKFILYDNGSDDGLEKVVEQLLLQGYDVKTLLWPWPKTQEAGFSHCAVYARDSCTWMMYVDVDEFVFSPSWLKSLHPSPDMLKSLLPLPRTSHGSSSSSSGLPIAQIMMRCLDFGPSNQTSHPIQGVTQGYTCRRKMEQRHKSIVLLEAVDTTLINVVHHFQLREGYRGKRLTRGEGVVNHYKYQAWSEFKAKFRRRVSAYVVDWRQESNPMSQDRTPGLGFAPIEPKGWATKFCEVNDSRLKVATQRWFRFGLDSPTGYKMAWQD

>RCOM16 Morus notabilis unknown function

MAQIMPLADPTLISLPTTHSSSSSSPLLRLSDSHNSSKQGLCHDAMRRRLRPNLFLTFAAIAFFLLLSLHLSRHAFSSQTTATDFLFSSSAAANLRSGNSDHYYNLAIHEDSDLLNNRHVRHVSSVKDSNSTAAASVSVLLTDWEVLVIVSHPVQPLSGDDFVCLFQNGDTSEARYSGVLPMNNRTTYKCTMPNSVRSLRPFFMPVLTKPSTEKEESPPPFQLLELYRWTFLVYESFSTEDDIVLFVKGVNNRQGRNRPPSDLNCVFFYDDGNNYNIATKTAVTSSAQEVFRCAPPELTSLDFNRPIKVSLEILYENMIVPSVAQYIRRCSLANPEPKSLLCAFAMVYNSGKFLKEWVTYYSKIGVDKFILYDNDSNDNLSSVVEELNGKGYNVTTLFWVWPKAQEAGFSHSAIYGKDSCSWMMYVDVDEFIYSPSWTSSSKPSDDMLKSLLPRAPPSKPSDGSINIGQVSIKCNDFGPSNQPTHPTAGVTQGYTCRRKAEERHKSILLLEAVDPSLLNVIHHFHLISDKYRSNTISMQRAVVNHYKYQAWSEFRNKFRRRVSTYVADWTEKVNPESKDRTPGLGFEAVEPKGWAKMFCEVRDERLKLLTRKWFGSRTSDGYKMAWENDDRDEE

>RCOM17 Cucurbita moschata unknown function

MRRKPCFAGLLLSCAVFFIFSFQISRKAFFSGGDLPSLSSDKLLPSRSTNSVAHYAIHEANLAHRNRQLSSVLRSIPTLSILLPDWEILLISSIHTPLSSPDSLRDFLCLFHNNATSPANFSGILDFTGRAKFKCRMPPSVRRLRPFFQPLLTKSPKNELSSSSSSPAMELMRWTFLAYESLETEDDVVLFVKGVNHRRGINRPPSDLKCVFGDGDDAIRTAVTSSEQEVFRCCHPNLTTRDDYNKMKITLEIFDSKGKSILVPSVAYYSPRYGGASLEAKSMICACTMVYNVGKFLKEWVIYYSSIGVEKFILYDNGSDDEISEIVKELKLEGYIIEIVFWIWPKTQEAGFSHSAEYSKKSCKWMMIVDIDEFVFSPSWLNSLEPSKNMLKSLIPPENNGIGMITIMCNDYGPSDRISHPAEGVTQGYNCRIKAEERHKSIVLLEAVDPSLLNVIHHFRLRKEFRWRKMKSSEAVVNHYKYQAWPEFRMKFRRRVSTYVVDWKDPANPTSKDRAPGLGNTAVEPPDWARKFCEVRDDRLRLLTRRWFGFQTAEGYRMAWQ

>RCOM18 Quercus suber unknown function

MRRRVRTAFLFVLLFTLVSVYLARNSITKKHVLNSTSDLRTRSKDPSFPNLAIRDDRERLDGQLRTRRVSYIGNSVATVSILFPDWEVFVIVSPEAALPSQDSGDAEYKCLFENGAKSPANFSGVLEFTNRTTFKCVLPNRLRSRRPFIQPVLIRSSETAVSPENKQVSAELIRWTTIAYESFSTENDVVLFVKGINRQLASKPPREFNCVFGDDGEKNTAVRTAVTSSNQEVFRCHHPKLTAAVTSERIKISLEIVPKNTVVPSVAYYTPRRRTLASQEPRSLLCACTMVYNVAKFLREWVMYHSRIGVEKFILYDNGSEDELAEVVDELNHEGFNVTTLFWIWPKTQEAGFSHAALYANQSCTWMMYVDVDEFVFSPTWQSRSSSKLLTSLLPISHRSIGQLQIRCNEFGPSNQTSHPVEGVTQGYTCRRKVDERHKSIVLLDAVDSSLENAIHHFEVRKSFFRSKLVSMEDAVVNHYKYQAWSEFRTKFRRRVSAYVVDWTRAVNPTSKDRTPGLGFEPIKPNGWAEKFCEVRDDRLKLLTQKWFGSNTSQGYKMAWQR

>RCOM19 Sorghum bicolor unknown function

MGQSPAMQPPSPRRRATTRALLRLLFLVALSLAALLAFVTSTPSSSFSTARRALQLVPPRRHRQPLTTLRAVRRDEDEPDRPTSPVVAPRAVGQGHHRSSDLDAVLLPDWEVLVLLRPGDDDDAPPGNATCAFPAGATSPARSLGRMPASGRRAYTCAMPRPERRHSRPFRAPRLVVVAATTTLSSSSSEEERSLTPEMMRWSGRLVYDSAALAAGGDVLVFAKGVNARQGVNRDATDVRCIYYRRGISGSAATVVASLPASTSAQQVFRCPPPPSTAAMTMTASPAEAQQLRVTIAVAGEDPIPSVATYTPPPPPPPPPPKKKLICGCTMVRDVAKFLREWVVYHAAVGVDRFYLYDNGSGDDLEGQVHQLSAEGFHVSTHAWPWPKTQEAGFSYTAAVHRDSCEWMAFIDVDEFIFSPDWAGSSKPTKSMLRSVVTAVKPNIGQVTLGCKDFGPSGRTKHPKEGVTQGYTCRRRAEERHKSLVRLDSVAPSLMNSVHHFKLRPEFKWERSRAVRVNHYKYQAWDEFKVKFRRRVSTYVADWTDRVNHGSKDRTPGLGFEAVEPAGWPHKFCEVEDTLLRDVTRRWFGVGFTNKLAHHRPVGGTTHSSS

>RCOM19-1 Quercus suber unknown function

MFSLKRSFQSTENPNFCLTHLPIFLYKSNPNSDTPTTALYFNLQEKMRRRVRTAFLFVLLFTLVSVYLARNSITKKHVLNSTSDLRTRSKDPSFPNLAIRDDRERLDGQLRTRRVSYIGNSVATVSILFPDWEVFVIVSPEAALPSQDSGDAEYKCLFENGAKSPANFSGVLEFTNRTTFKCVLPNRLRSRRPFIQPVLIRSSETAVSPENKQVSAELIRWTTIAYESFSTENDVVLFVKGINRQLASKPPREFNCVFGDDGEKNTAVRTAVTSSNQEVFRCHHPKLTAAVTSERIKISLEIVPKNTVVPSVAYYTPRRRTLASQEPRSLLCACTMVYNVAKFLREWVMYHSRIGVEKFILYDNGSEDELAEVVDELNHEGFNVTTLFWIWPKTQEAGFSHAALYANQSCTWMMYVDVDEFVFSPTWQSRSSSKLLTSLLPISHRSIGQLQIRCNEFGPSNQTSHPVEGVTQGYTCRRKVDERHKSIVLLDAVDSSLENAIHHFEVRKSFFRSKLVSMEDAVVNHYKYQAWSEFRTKFRRRVSAYVVDWTRAVNPTSKDRTPGLGFEPIKPNGWAEKFCEVRDDRLKLLTQKWFGSNTSQGYKMAWQR

>RCOM20 Cannabis sativa unknown function

MGMGGKTLIIPITNKLLLIITSLFLFVLLSLHLSRHHILVTTHLDFPYTTTSKLNVAATPKYNAAAEEEENESQPILRNVSSPLHHHSVLLPEWEVLLIVPGPVNKETIDLFADYICLFQNNATSPATFSGALSNDRFAFKCSMPNSVRRLRPFLQPIMMTKSSEKESAAIVKMPELYRWNFLVYESFSTENDVVLFAKGINNRQWISRSPREFNCVFYHDSSKTVAFKTAVTSSAQEVFRCPHPNITAPFNSNDDYDDEDNGELKIKVSIEIISENNLVVPSVAYYGPRQIPQSKSKSELCASTMVYNAGKFLKEWVVYHSKIGVDKFILYDNDSNDNIKSVVEELNDEGFDVTTIFWVWPKSQEAGFSHSAIQSKDLCNWMMFVDVDEFIYSPSWFNFSKPSKSMLQSLLPTFSPSGEPNYIGQISIRCNEFGPSSQRSHPPEGVTQGYTCRRSVEQRHKSIVLLDAVNTSLLNAIHHFKMKDRYKTVTMSLNRAVVNHYKYQAWSEFQNKFRRRVSTYVVDWKEKANLASNDRTPGLGFEAVEPKAWDQKFCEVKDERLKLVTRKWFGSPTSDGYKLVWQT

>RCOM21 Juglans regia catalyzes the committed step in the biosynthesis of hydrolysable tannins and biosynthesis of nonstructural phenols(The walnut (Juglans regia) genome sequence reveals diversity in genes coding for the biosynthesis of non-structural polyphenols)

MRPRVRKALLSCFLAIVLFAFFSVRLSRKAISVATVRYSTSNLTVRSKINPNSLNYAVQENHHDRDAQELSRPRTRHVSSIWDSIATLSVLLPAWEVLVIVPPETPLSPGLGEEYRCIFQNNATSTATFSGVLAFTNRTTFKCVMPDSVRRLRPFLQPILIKQSENESPVTKPMPELIRWTFMAYESFSTEDDVILFVKGVNHRQGLNRSPQEFLCLFGDGTTSFVKTAVTSSIQEVFRCPHPNLMGVISGADSERIKISLEIVTENLVVPSVAYYTPRRSLVIGRPEEPRSILCACTMVHNVAKFLKEWVVYHSMIGVQKFILYNNDSDDDLERVVEELNQDGYNVTTLFWIWPKTQEAGFSHGAVYAKDSCTWMLYVDVDEFVFSPSWDQFKKPSNDMLTSLLSRTPQKTPSSSSLMLPASHRRIGQVVMRCNEFGPSNQTSHPAEGVTQGYTCRRKIEQRHKSMVLLDAMDPSLLNVIHHFQLKEGFRSKTMSMEDAVVNHYKYQAWSEFRTKFRRRVSAYVVDWMQAVNPKSKDRTPGLGFEPIEPKGWAQKFCEVRDERLKLLTQKWFGSTEY

>RCOM22 Cucurbita maxima unknown function

MRRKPCFAGLLLSCAVFLIFSFQISRKAIFSGGDLPSLSSDKLLPSRSKNSVAHYAIHEANLAHRNRQLSSVLGSIPTLSLLLPDWEILLISSIHTPLSSPDSLRDFLCLFHNNATSPANFSGVLDFTGRATFKCRMPPSVRRLRPFFQPLLTKSPKNELSSSSSSPAMELMRWTFLAYEALETEDDVVLFVKGVNHRRGINRPPSDLKCVFGDGYDAIRTAVTSSEQEVFRCRHPNLTTRDDYEKMKITLEIFDAKGKSILVPSVAYYSPRYGGDSVEAKSMICACTMVYNVGKFLKEWVIYYSRIGVEKFILYDNGSDDEISEIAKELKLEGYIIEIVFWIWPKTQEAGFSHSAEYSKKSCKWMMIVDIDEFVFSPSWLNSLEPSKNMLKSLIPAEKNGIGMITIMCNDYGPSDRISHPAEGVTQGYNCRIKAEERHKSIVLLEAVDPSLLNVIHHFRLRKEFRWRKMKSSEAVVNHYKYQAWPEFQMKFRRRVSTYVVDWKDSANPTSKDRAPGLGNSAVEPPDWPRKFCEVRDDRLRLLTRRWFGFQTAEGYRMAWQ

>RCOM23 Benincasa hispida unknown function

MRRKPRSTGLLFSVAVFLLFAFQFSRKAFFNGADLPFPSSYNLLPSRSANSEAHYALHETNIDFPHQLTHRTRHVSSILDPVPTVSLLLPDWEVLLISSIDTPLASPDSLRDFLCLFQNNATSSANFSGVLDFTGRLTFRCFMPESVRRLRPFFQPLLIKSPDKEFSSYSSSSLAPELMRWTFFAYEAFETEDDVVLFVKGVNHRQGNNRPPTDLNCVFGDGDNAVRTAVTSSVQEVFRCRHPNLTTREDHDKFKITLEILDKGKSVIVPSVAYYSPRRSDGGGGSLAAQSMICACTMVYNVGKFLKEWVMYYSRIGVDKFILYDNGSDDEISAVVKELKQEGYNIEIVFWIWPKTQEAGFSHSVEYSKKSCKWMMFVDIDEFVFSPSWLNSLKPSKNMVKSLLPAENNGIGMITVMCNDYGPSDRISHPTEGVTQGYNCRRKLEERHKSIVLLEAVDRSLLNVIHHFKLKTEFRSRQMRPEEAVVNHYKYQAWPEFRMKFRRRVSAYVVDWKDSANPTSKDRAPGLGNTAVEPPDWPRKFCEVRDDRLRLLTQRWFGSQTADGYRMAWQ

>RCOM24 Diospyros lotus unknown function

MLEEESLHFRISYTFSPIRSMAQNVRKGFIAVLATIIFLASFYHSLLAPKPPFSAADLRAPPIPRFPRTDANSVIQEQEQPKHEPVSRQIISRHVTSVIDSINTVSILLPAWEVLVIISPKNRFLSDSVPTGFTCLFPSNASSPGKFAGFLSSPRRSAFKCDFPGRLRRRLPFPQPVLSRSPDYPPEIPFPAPELIRWNYLVYESFSTETDVVLFVKGINHRQGVNRAPSEFRCVFGDDDGINAVKTAVTTSLQEVFRCPNPPLVTSAFLSNGRIKISLEILQQKRIVPSVAYYTSSRKIALQQTKSLICACTMVYNVAKFLKEWVVYYSAIGVEKFILYNNGSDDQLDRIVEELVSEGYNVSTLFWLWPKTQEAGFSHCAISAKDSCEWMIYMDVDEFVYSPSWLNSSTPSTQMLNDLLPESSLFGQILISCYEFGPSNQNSHPELGVTQGYNCRRRSENRHKSIVLLEAIDESLLNTVHHFQLKQGYIGKRLGVKEAVVNHYKFQAWSEFKTKFRRRVSAYVVDWTVELNLASKDRTPGLGSAPVEPPAWAEMFCEVYDEQLKELTRRWFGSESPSGYGMAWQR

>RCOM25 Lolium perenne unknown function

MQLHARRRHAVARCRALITAAAFALALAALLCFSLVPPGGMLAAPARALQLVPARRLHVRLGAVKGYTTTATATPAAGGRATKAQPDAAVLLPDWEALVLLRADAADAAGNVTCRFRGGATSPARALGRLPAPGRSGRRAYACAVPEPARRHKTLPAPQLVIASSSTSKRTVGAARGGGRSTEMLRWSGRLVYESAVVDGGDVLVFAKGVNPRQGVNRPASDIQCVYYRAGDHVVATLPAATSAQQVFRCPPPPTPVQQESQEEIRVTLAVTGQEPLPSLAVYNPPRAASSTAPEKKLICACTMVRDVAKFLPEWVVYHAAVGVDRFYLYDNGSEDDLADQVHHLNAAGYDVSTVAWPWTKAQEAGFSHSAAAHRHSCQWMAFVDVDEFIFSPQWNQSNNPDKSTMLRSVVSSAEPDVGQVSLGCADFGPSGQTSNPEEGVTQGYTCRRRTQERHKSLLRLDAVDDSLVNSIHHFALRPGFQGQWNKQVRVNHYKYQAWEEFKVKFRRRVSTYVADWTDPVNLKSKDRTPGLGFEAVEPVGWTHKFCQVNDTLLRDATRRWFGAGFRNGPARRQTGTSSSS

>RCOM26 Citrus unshiu unknown function

MGYQRALSVSFSLRDDEYHTTSRRTRTRFSRLFFSLLLLAAFSFYVSSAISGSSFYFFQVNLTVQSNHVTNQGFEELYNVTSLSRHVSSVEEDSVHKYDSVSVLLPDWEVLVILSPENPLMPSDPLEGFHCLFSNNQTSPARFSGALPFTERTAFKCIMPNGVRRQMPFWQPILTKHPEKETLPKEEQRELMNMKNLAYESISTESDVVLFVKGVNSRSGINSPPQDFMCIFGDDVVHGTVKTHVTSSMQEVFRCSHPDLTALSSCGADQGRPIKISLQITKENRTVPSVAYYSPRHEIALTQEPKKSEICACTMVYNVGKFLKEWVIYHSKIGVDKFILYDNGSDDGLQTVVNELNLDGYDVTTLLWLWPKTQEAGFSHSAIHAKDSCKWMLYVDVDEFVYSPSWEKASQPSKHMLKSLLPQQPSSIGQVSFRCNDFGPSGQKSHPVRGVTQGYNCQRRVKEPRHKSIVLLEAIDDSLGNVVHHFGLKKTFKWKHMSVHDGVVNHYKYQAWSEFKVKFRRRVSAYVADWKEKTNPNSKDRTPGLGFQPIEPEGWEHKFCDHRDDRLKLLTQRWFGEQTPHGYKMAWQR

>RCOM27 Citrus clementina unknown function

MGYQRAHSVSFSLRDDEYHTTSRRTRTRFSQLFFSLLLLAAFSFYVSSAISGSSFYYFQVNLTVQSNHVTNQGFEELYNVTSLSRHVSSVEEDSVHKYESVSVLLPDWEVLVILSPENPLMPSDSLEGFHCLFSNNQTSPARFSETLPFTERTAFKCIMPNGVRRQMPFWQPILTKHPEKETLPKEEQRELMNMKNLAYESISTESDVVLFVKGVNSRSGINSPPQDFMCIFGDDVVHGAVKTPVTSSMQEVFRCSHPDLTALSSSGADQGRPIKISLQITKENRTVPSVAYYTPRHEIALTQEPKKSEICACTMIYNVGKFLKEWVIYHSKIGVDKFILYDNGSDDGLQTVVNELNLDGYDVTTLLWLWPKTQEAGFSHGAIHAKDSCKWMLYVDVDEFVYSPSWDKASQPSKHMLKSLLPQQSSSIGQVSFRCNDFGPSGQKSHPVRGVTQGYNCQRRVKEPRHKSIVLLEAIDDSLGNVVHHFGLKKTFKWKQMSVHDGMVNHYKYQAWSEFKAKFRRRVSAYVTDWKEKTNPNSKDRTPGLGFQSIEPEGWEHKFCDHRDDRLKLLTQRWFGEQTPHGYKMAWQR
